# Development of Potent and Selective Inhibitors of Methylenetetrahydrofolate Dehydrogenase 2 for Targeting Acute Myeloid Leukemia: SAR, Structural Insights, and Biological Characterization

**DOI:** 10.1021/acs.jmedchem.4c01775

**Published:** 2024-11-26

**Authors:** Hsin-Huei Chang, Lung-Chun Lee, Tsu Hsu, Yi-Hui Peng, Chih-Hsiang Huang, Teng-Kuang Yeh, Cheng-Tai Lu, Zih-Ting Huang, Ching-Cheng Hsueh, Fang-Chun Kung, Li-Mei Lin, Yu-Chen Huang, Yi-Hsin Wang, Li-Hsuan Li, Ya-Chu Tang, Ling Chang, Chih-Chien Hsieh, Weir-Torn Jiaang, Ching-Chuan Kuo, Su-Ying Wu

**Affiliations:** † Institute of Biotechnology and Pharmaceutical Research, National Health Research Institutes, 35 Keyan Road Zhunan Town, Miaoli County 350, Taiwan, Republic of China; ‡ Institute of Biotechnology, National Tsing Hua University, Hsinchu 300, Taiwan, Republic of China

## Abstract

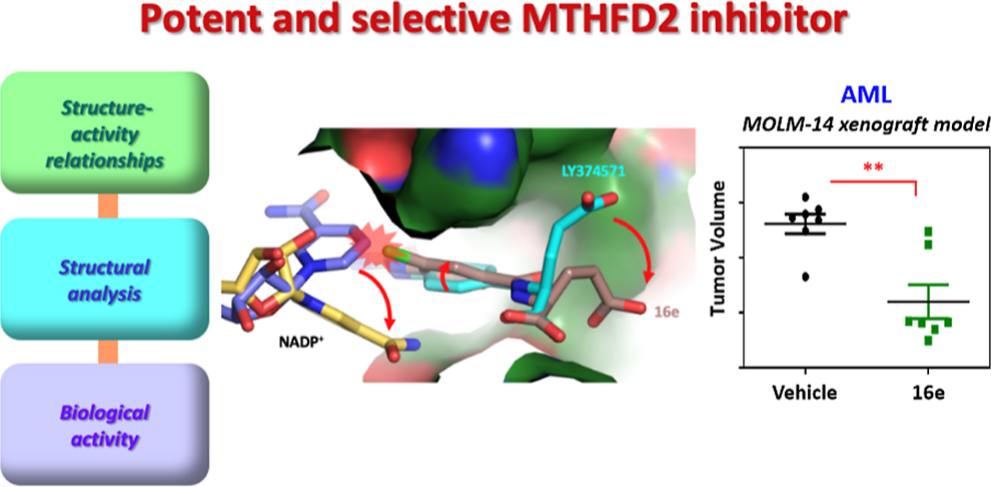

Methylenetetrahydrofolate dehydrogenase/cyclohydrolase 2 (MTHFD2), a pivotal mitochondrial enzyme in one-carbon metabolism, is significantly upregulated in various cancers but minimally expressed in normal proliferating cells. In contrast, MTHFD1, which performs similar functions, is predominantly expressed in normal cells. Therefore, targeting MTHFD2 with selective inhibitors holds promise for a broader therapeutic window with reduced toxicity and fewer side effects. This study identified selective 2,4-diamino-6-oxo-1,6-dihydropyrimidin-5-yl ureido-based derivatives through systematic chemical modifications and SAR studies. Structural biology investigations revealed substitutions in the phenyl ring and tail region modulate potency and selectivity toward MTHFD2. Additionally, a comprehensive cell screening platform revealed acute myeloid leukemia cells with FLT3 internal tandem duplication mutations are particularly sensitive to these inhibitors. Furthermore, synergistic effects were observed when combining potential compounds with Alimta. Compound **16e** emerged as a leading candidate, demonstrating superior inhibition and selectivity for MTHFD2, favorable pharmacokinetics, and potent antitumor efficacy in MOLM-14 xenograft models.

## Introduction

**Figure 1:**
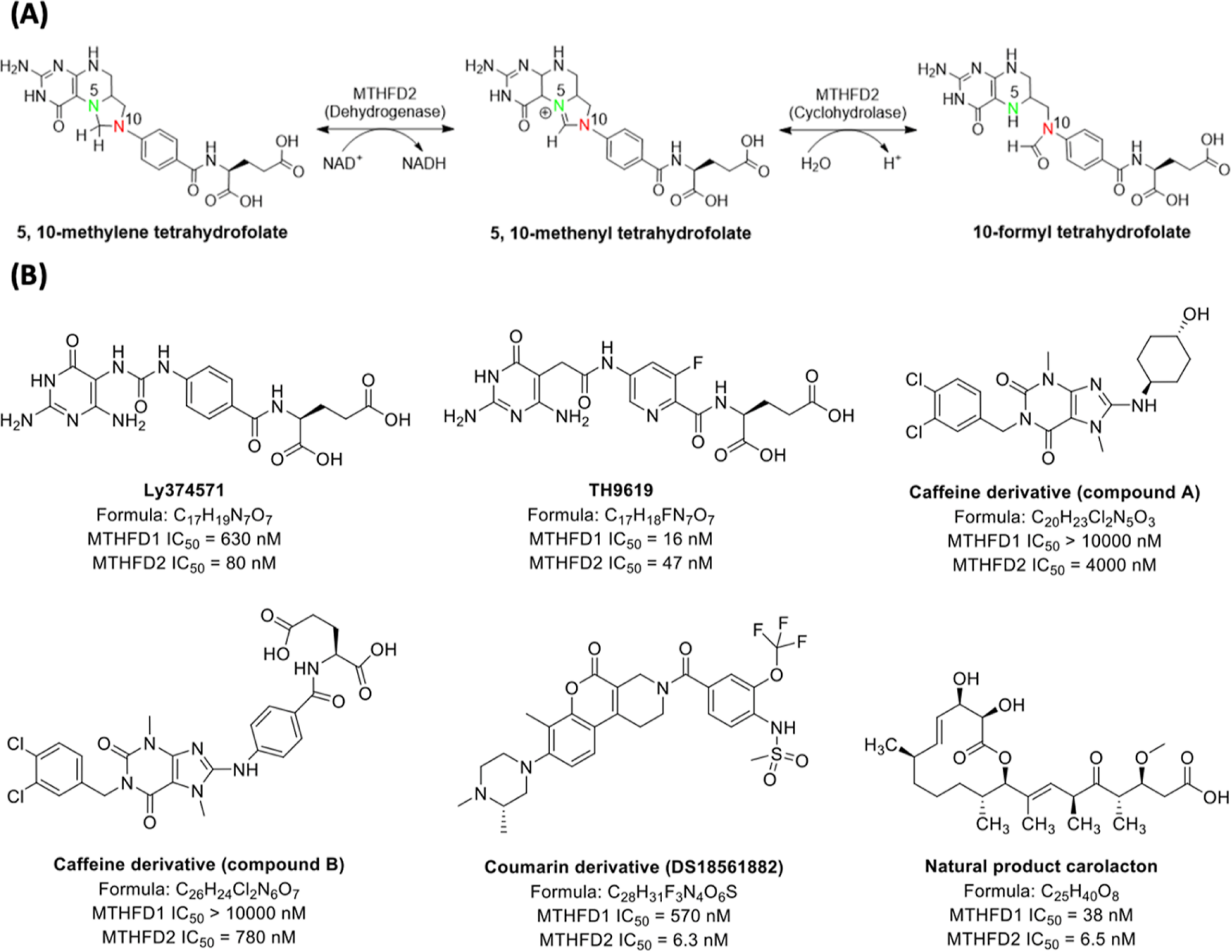
(A) Catalytic reaction of MTHFD2. (B) Structures of MTHFD2 inhibitors.

Methylenetetrahydrofolate dehydrogenase/cyclohydrolase 2 (MTHFD2) is a bifunctional metabolic enzyme found in mitochondria, exhibiting both dehydrogenase and cyclohydrolase catalytic activities.^1,2^ In one-carbon metabolism, MTHFD2 converts 5, 10-methylene tetrahydrofolate (5,10-CH_2_-THF) to 10-formyl tetrahydrofolate (10-CHO-THF) in mitochondria (Figure 1A), thereby regulating the production of NADH, purines, and pyrimidines.^1,3^ MTHFD2 has been discovered as one of the most overexpressed metabolic genes in cancer cells.^4^ Numerous studies have shown that the knockdown of MTHFD2 impairs tumor growth in various cancers, including colorectal cancer,^3,5^ ovarian cancer,^6−8^ breast cancer,^4,9−11^ nonsmall cell lung cancer (NSCLC),^12,13^ and acute myeloid leukemia (AML).^14,15^ These findings suggest that MTHFD2 holds significant potential as a therapeutic target for cancer treatment. By inhibiting MTHFD2, it would be feasible to strategically disrupt the metabolic pathways crucial for sustaining tumor growth and proliferation, offering a promising approach for the development of novel cancer therapies.^1,16−19^

An isozyme, methylenetetrahydrofolate dehydrogenase, cyclohydrolase and formyltetrahydrofolate synthetase 1 (MTHFD1), performs similar catalytic reactions to MTHFD2, albeit localized in the cytoplasm rather than the mitochondrion. Moreover, MTHFD1 possesses additional enzymatic activity as a 10-formyl tetrahydrofolate synthetase, converting 10-CHO-THF to formate.^1,20^ The key distinction between these two proteins lie in their expression profiles: MTHFD1 is typically expressed in normal cells while MTHFD2 is abundantly expressed in immortal,^21^ embryonic,^22^ and cancer cells,^4^ but absent in adult normal cells.^4^ Therefore, designing inhibitors specifically against MTHFD2 holds promise for achieving a broader therapeutic window with minimized toxicity and fewer side effects.

Several MTHFD2 inhibitors have been identified (Figure 1B). LY374571, a 2,4-diamino-6-oxo-1,6-dihydropyrimidin-5-yl ureido based derivative, was originally developed by Eli Lilly and its chemical structure was subsequently re-evaluated by Eadsforth et al.^23,24^ Our study found it concurrently inhibited both the isoforms of MTHFD1 and MTHFD2 with IC_50_ values of 630 and 80 nM, respectively. TH9619, an analogue of LY374571 reported by Bonagas et al., showed potent inhibition against MTHFD2 with an IC_50_ value of 47 nM.^15^ TH9619, administered at 60 mg/kg four times daily, prolonged mouse survival on a low-folate diet with intravenously injected HL-60 xenograft tumors. Raze Therapeutics discovered caffeine (xanthine-based) inhibitors of MTHFD2, exemplified by compound A, which showed selectivity to MTHFD2.^25,26^ Further structural modification of compound A, as reported by Lee et al., revealed that compound B allosterically binds to MTHFD2 and coexists with the substrate analogue.^26^ A series of tricyclic coumarin-based derivatives incorporating a sulfonamide group was disclosed by Kawai et al. as MTHFD2 inhibitors. DS18561882, the most promising compound in the coumarin series, was an orally available MTHFD2 inhibitor and demonstrated antitumor effects in an MDA-MB-231 mouse xenograft model.^27^ Moreover, carolacton, a natural product, exhibited low-nanomolar inhibition against both human MTHFD1 and MTHFD2.^28^

Several structurally diverse inhibitors of MTHFD2 have been identified; however, only a few crystal structures of MTHFD2 in complex with inhibitors have been reported. The first is that of human MTHFD2 in complex with LY345899, NAD^+^ and phosphate.^29^ MTHFD2 forms a homodimer, with LY345899 occupying the substrate binding site. The cofactor NAD^+^ was bound to the C-lobe, where the phosphate group interacted with the hydroxyl group of NAD^+^ and formed an extensive network in the dimer interface. Recently, the same group reported structures of inhibitors TH7299, TH9028, and TH9619 bound to MTHFD2.^15^ Kawai et al. reported structures of the MTHFD2 in complexed with tricyclic coumarin derivatives.^27,30^ The coumarin derivatives bound within the substrate-binding site, albeit with a binding mode that differed slightly from LY345899. Unlike LY345899, which formed a hydrogen bond network through its pteridine moiety, the interactions between the coumarin scaffold and its terminal moiety with the surrounding residues contributed to the inhibitory effect of these inhibitors. We previously solved the crystal structures of MTHFD2 in complex with the xanthine derivatives.^26^ Compound B was identified as an allosteric binder to MTHFD2, leading to significant conformational changes in the protein. This binding event also hindered the binding of the cofactors NAD^+^ and phosphate to MTHFD2.

In this study, we report the synthesis, chemical structural modification and systematical structure–activity relationship (SAR) study that led to the identification of selective 2,4-diamino-6-oxo-1,6-dihydropyrimidin-5-yl ureido based derivatives. To elucidate their binding mechanism and selectivity, we determined the protein structures of MTHFD2 complexed with 2,4-diaminopyrimidine-based derivatives, alongside those of MTHFD1. Comparative analysis revealed that additional substitutions on the phenyl ring of these inhibitors caused the shift in the nicotinamide group of cofactor NADP^+^ and induced conformational changes in the phenyl ring and γ-carboxylic acid of the inhibitors, thereby disrupting their interactions with MTHFD1. Furthermore, we present the first systematic establishment of a cell screening platform for MTHFD2 inhibitors using a diverse panel of leukemia and solid tumor cell lines. Our findings showed that AML cells with FLT3 internal tandem duplication (ITD) mutations, an alteration in the FLT3 gene encoding a tyrosine kinase involved in cell growth and survival signaling pathways, exhibited the most favorable responses to this series of MTHFD2 inhibitors. Moreover, a promising candidate, **16e**, with high potency and selectivity to MTHFD2, demonstrated excellent pharmacokinetics profiles and exhibited in vivo antitumor efficacy. These findings would provide insights and hold promise for advancing cancer research by targeting MTHFD2.

## Results and Discussion

### Chemistry

**Scheme 1:**
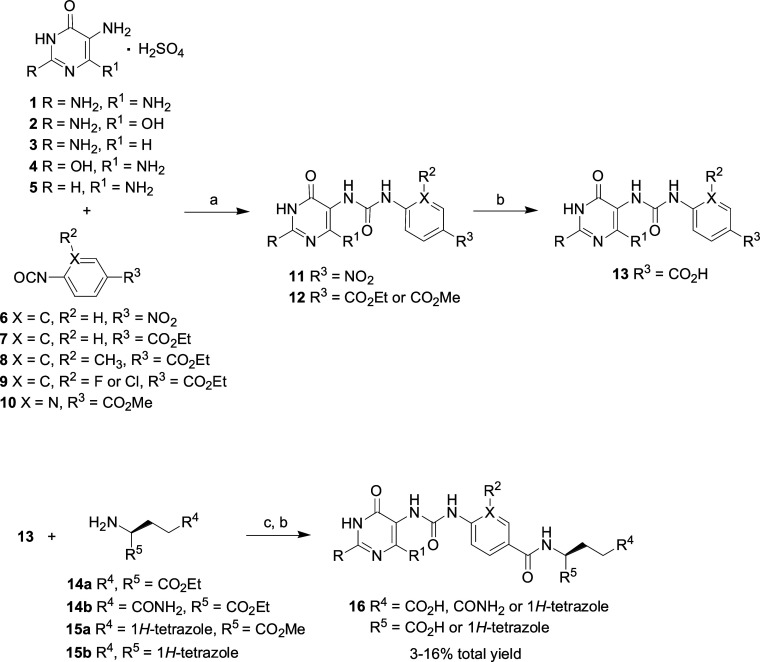
Reagents and Conditions: (a) 1 N NaOH, CH_3_CN, 25 °C; (b) 1 N NaOH, H_2_O, 25 °C for Ester Compounds **12**, **14a**, **14b** and **15a**; (c) EDC HCl, HOSu, Dry DMSO, 25 °C

**Scheme 2:**
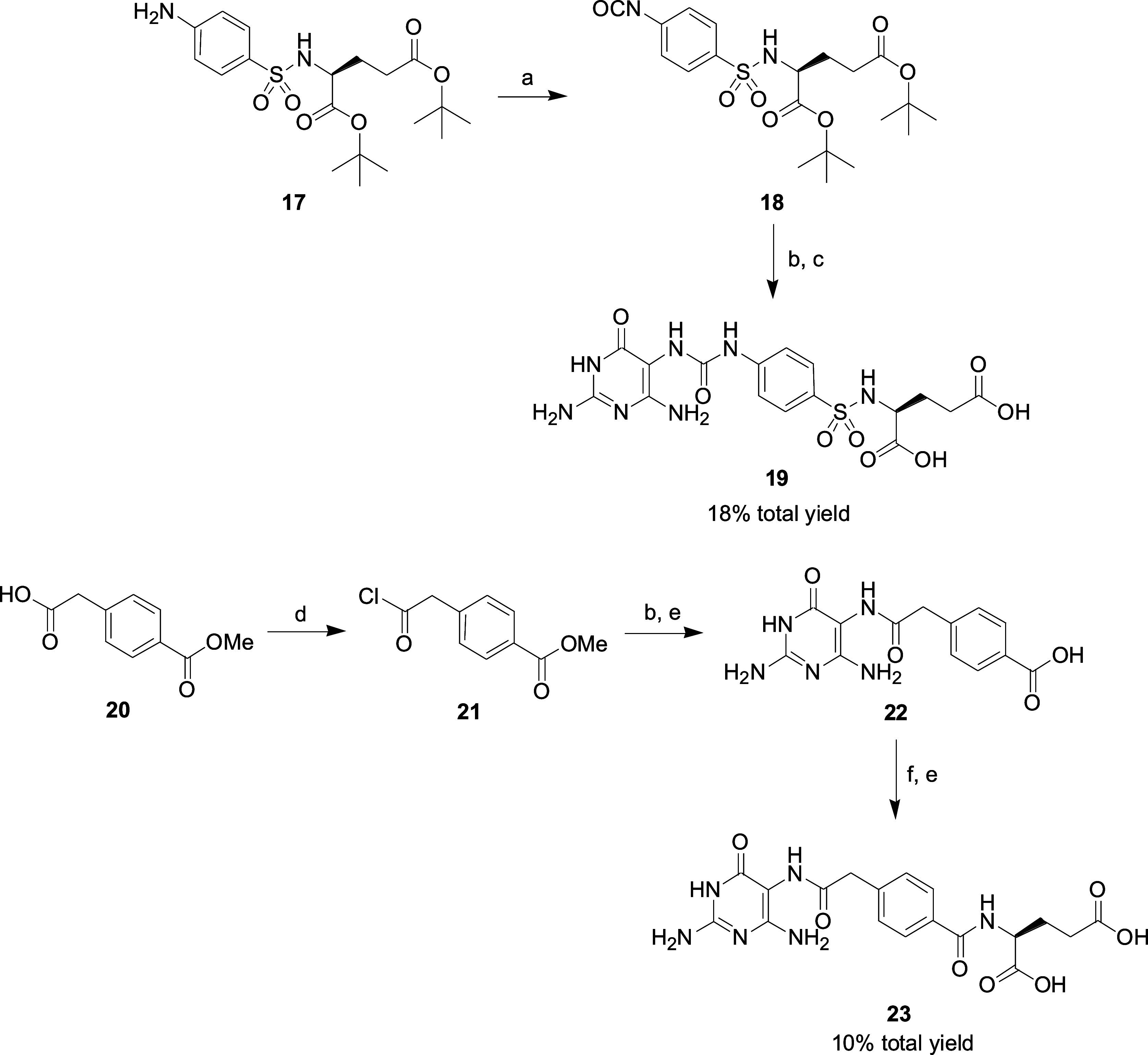
Reagents and Conditions: (a) bis(Trichloromethyl)carbonate, CH_2_Cl_2_, DIPEA, 15 °C; (b) Compound **1**, 1 N NaOH, CH_3_CN, 25 °C; (c) TFA, 25 °C; (d) SOCl_2_, Pyridine, CH_2_Cl_2_, 55 °C; (e) 1 N NaOH, H_2_O, 25 °C; (f) l-Glutamic Acid Diethyl Ester Hydrochloride, EDC HCl, HOSu, dry DMSO, 25 °C

The general synthetic routes to the final products **11**, **16**, **19** and **23** derived from LY374571 can be prepared according to previously described methods^15,31^ and are shown in Schemes 1 and 2. 2,5,6-Triamino-3*H*-pyrimidin-4-one sulfate **1**, 2,5-diamino-6-hydroxy-3*H*-pyrimidin-4-one **2**, 2,5-diamino-3*H*-pyrimidin-4-one **3**, 5,6-diamino-2-hydroxy-3*H*-pyrimidin-4-one **4** or 5,6-diamino-3*H*-pyrimidin-4-one **5** in NaOH solution was reacted with 1-isocyanato-4-nitrobenzene **6**, ethyl 4-isocyanatobenzoate **7**, 4-isocyanato-3-methylbenzoate **8**, 4-isocyanato-3-florobenzoate **9** or methyl 6-isocyanatonicotinate **10** in CH_3_CN to provide the urea **11** or **12**. The hydrolysis of ester group (**12**) was catalyzed by 1 N NaOH solution to give acid **13**. Condensation of acids **13** with l-glutamic acid diethyl ester hydrochloride **14** or 2-amino-4-(1*H*-tetrazol-5-yl)butyric acid methyl ester **15**(32) using coupling reagents EDC HCl (1-ethyl-3-(3-(dimethylamino)propyl)carbodiimide hydrochloride) and HOSu (*N*-hydroxysuccinimide) followed by hydrolysis of the methyl ester group by NaOH produced target compounds **16** (Scheme 1).

Compound **17** obtained by a known procedure^33^ was reacted with bis(trichloromethyl)carbonate to form the corresponding isocyanate **18**. Compound **1** was coupled with isocyanate **18** and then deprotected the *tert*-butyl ester group by TFA to yield the desired benzenesulfonylamino **19**. The reaction of SOCl_2_ with 4-carboxymethylbenzoic acid methyl ester **20** in the presence of pyridine gave acyl chloride **21**. The synthetic route for the preparation of the final product diacid **23** starting from **21** is like that of **16** (Scheme 2).

### SARs of Diaminopyrimidine-Based Derivatives

Compound LY374571, originally discovered by Eli Lilly^23^ and the chemical structure later correctly determined by Eadsforth et al.,^24^ has been chosen as the lead compound for the development of selective MTHFD2 inhibitors. LY374571 was a competitive inhibitor of human MTHFD1 (IC_50_ = 630 nM) and MTHFD2 (IC_50_ = 80 nM). To enhance its inhibitory potential and selectivity, a systematic structure–activity relationship study was conducted, starting from l-glutamic acid moiety of LY374571 (Table 1). Removal of glutamic acid moiety of LY374571 lost the inhibitory activity against MTHFD1 and MTHFD2 (**11**, IC_50_ > 10 μM). Bioisosteric replacement of γ-carboxylic acid of glutamic acid with 1*H*-tetrazole to give **16a** significantly increased the potency against MTHFD1 (IC_50_ = 120 nM) and MTHFD2 (IC_50_ = 22 nM). However, the selectivity index (SI), calculated as the ratio of the IC_50_ of MTHFD1 to the IC_50_ of MTHFD2, of **16a** was slightly decreased (SI = 5.5) compared to LY374571 (SI = 7.9). When α- and γ-carboxylic acid of glutamic acid was replaced with 1*H*-tetrazole, compound **16b** exhibited moderate inhibition against MTHFD2 (IC_50_ = 150 nM) with low selectivity over MTHFD1 (SI = 2.5). Furthermore, the replacement of the glutamate tail (LY374571) with a glutamine tail (**16c**) resulted in decreased inhibition against MTHFD2 and reduced selectivity. The replacement of the carboxyl group next to the central phenyl ring in LY374571 with a sulfonyl moiety (**19**) led to the complete loss of inhibitory activity against MTHFD1 and MTHFD2 (IC_50_ > 10 μM), even when the glutamate tail was retained. Next, the substituent effects at the third position of phenyl ring were also examined. Methyl substituted **16d**, chloro substituted **16e** and fluoro substituted **16f** had minor effects on the inhibition of MTHFD2 with IC_50_ values ranging from 66 to 134 nM. However, larger methyl and chloro substituents (**16d** and **16e**) exhibited higher selectivity indices (SI) of 38.5 and 27.1, respectively, compared to the unsubstituted (LY374571, SI = 7.9) and fluoro-substituted (**16f**, SI = 6.6) analogues. In comparison, 1*H*-tetrazole **16g** with a 3-chloro substituent on the phenyl ring does not improve selectivity (SI = 5.8) when compared with unsubstituted analogue **16a** (SI = 5.5). Furthermore, when the central linker of LY374571 was changed from the phenyl ring to the pyridine group, **16h** showed decreased inhibition against MTHFD1 (IC_50_ = 4140 nM) and MTHFD2 (IC_50_ = 470 nM).

**Table 1:**
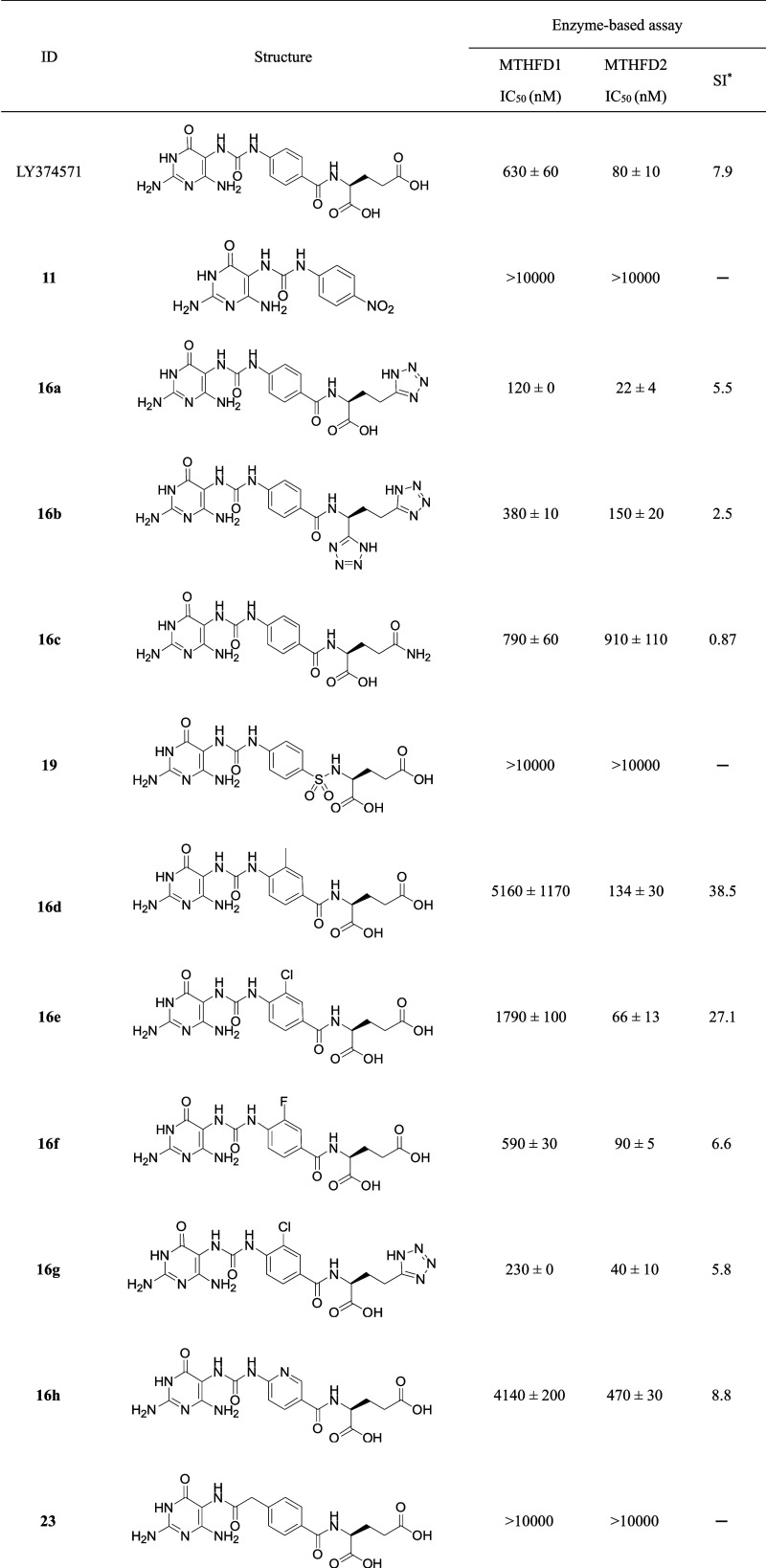

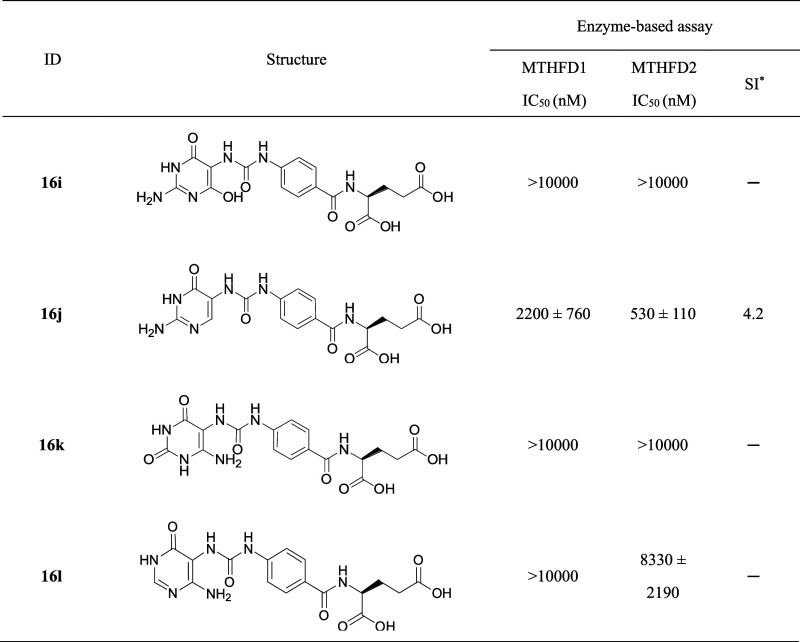
Inhibition of MTHFD1 and MTHFD2 with Diaminopyrimidine-Based Derivatives

a SI, the ratio of IC_50_ for MTHFD1/IC_50_ for MTHFD2.

In addition to evaluating the effects of the terminal amino acids and substituents at the third position of central phenyl ring, the urea linker and triaminopyrimidine headgroup of LY374571 were also modified. Replacement of the urea moiety with an amide group (**23**) resulted in complete loss of inhibitory activity. Subsequently, the effects on replacing the headgroup, 2,5,6-triamino-3*H*-pyrimidin-4-one ring, on LY374571 with alternative pyrimidine groups, such as 2,5-diamino-6-hydroxy-3*H*-pyrimidin-4-one (**16i**), 2,5-diamino-3*H*-pyrimidin-4-one (**16j**), 5,6-diamino-1*H*-pyrimidine-2,4-dione (**16k**) and 5,6-diamino-3*H*-pyrimidin-4-one (**16l**), were evaluated. Replacement of NH_2_ group at the sixth position of 2,5,6-triamino-3*H*-pyrimidin-4-one (LY374571) with OH group to yield **16i** completely lost the activities of MTHFD1 and MTHFD2 (IC_50_ > 10 μM). Removing the NH_2_ group at the sixth position of 2,5,6-triamino-3*H*-pyrimidin-4-one (**16j**) decreased the inhibition of MTHFD1 and MTHFD2, with IC_50_ values of 2200 and 530 nM, respectively. Further structural modifications at the second position of headgroup of LY374571 to give **16k** (2-one) or **16l** (2-H) resulted in a dramatic loss of potency against both MTHFD1 (IC_50_ > 10 μM) and MTHFD2 (IC_50_ > 10 μM and IC_50_ = 8330 nM, respectively).

### Structure Biology Studies of Diaminopyrimidine-Based Derivatives

**Figure 2:**
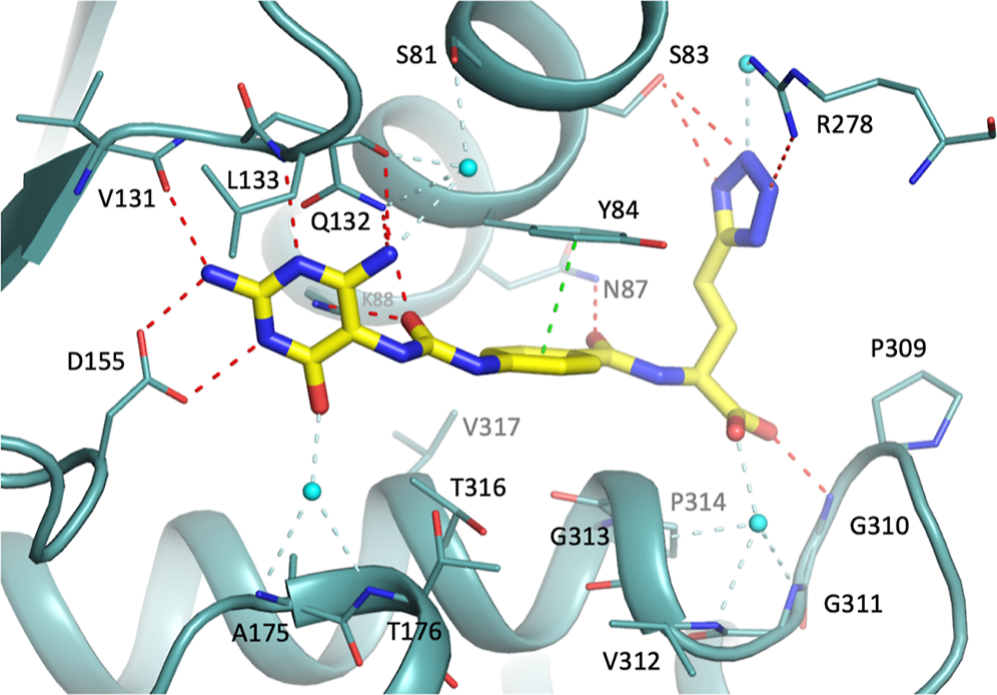
Crystal structure of MTHFD2 (light teal) in complex with **16a** (yellow) and NAD^+^ (omitted for clarity) (PDB: 9ISC). Hydrogen-bond with protein, water -bridged and π–π interactions are shown in red, pale-cyan and green dashed lines, respectively. Water molecules are shown as spheres (cyan). For clarity, the NAD^+^ is omitted in the figure.

In order to investigate the binding mechanisms of MTHFD2 inhibitors and gain insights into their interactions with MTHFD2, structural biology studies were conducted to determine the structure of human MTHFD2 in complex with **16a**, as well as compounds **16d**, **16e**, **16g**, and LY374571. These investigations were conducted under conditions that included the presence of NAD^+^ and phosphate, and the resulting cocrystal structures were resolved at a resolution ranging from 2.04 to 2.54 Å. Data collection and refinement statistics are summarized in Table 2. The structure of MTHFD2/**16a** (Figure 2) revealed the headgroup, 2,5,6-triamino-3*H*-pyrimidin-4-one ring of **16a**, establishes a hydrogen bond network with key amino acid residues, including Val131, Leu133, and Asp155. The carbonyl group of the triaminopyrimidine was hydrogen bonded with the main chain of Ala175 and Thr176 through a water molecule. Additionally, hydrophobic interactions occurred between the triaminopyrimidine headgroup and Leu130 and Phe157. The urea moiety of **16a** formed hydrogen bonds with the side chain of Lys88 and Gln132. In the middle region of **16a**, the phenyl ring formed robust π–π interactions with Tyr84, and hydrophobic interactions with neighboring amino acids such as Tyr84, Ile276, Thr316, and Val317. The amide moiety adjacent to the phenyl ring was hydrogen bonded with Asn87 and participated in hydrophobic interactions with Gly313.

**Table 2:** X-ray Data and Structure Refinement for MTHFD2 Complex Structures

	MTHFD2/LY374571	MTHFDD2/**16a**	MTHFD2/**16d**	MTHFD2/**16e**	MTHFD2/**16g**
resolution	28.90–2.12	27.18–2.54	27.26–2.35	28.91–2.06	28.62–2.04
space group	*P*6_5_	*P*6_5_	*P*6_5_	*P*6_5_	*P*6_5_
unit cell *a* = *b*, *c* (Å)(α = β = 90°, γ = 120°)	115.61, 113.33	115.56, 112.96	115.34, 113.36	115.62, 113.27	114.85, 113.41
unique reflections	48918 (4856)	28338 (2823)	35675 (3516)	53096 (5290)	54200 (5369)
*I*/σ	20.45 (2.18)	13.9 (2.14)	16.53 (2.13)	24.48 (3.21)	19.63 (2.06)
Rmerge (%)	6.1 (50.9)	6.5 (37.6)	7.9 (62.4)	5.7 (52.4)	6.2 (47.3)
completeness (%)	99.7 (100.0)	98.0 (99.8)	99.8 (99.9)	99.3 (100.0)	99.3 (99.8)
Rwork/Rfree	0.2084/0.2414	0.1919/0.2397	0.2068/0.2488	0.2111/0.2392	0.1965/0.2237
r.m.s.d (bond) (Å)	0.007	0.008	0.008	0.008	0.007
r.m.s.d (angle) (deg)	0.931	1.037	1.024	0.942	0.917
Ramachandran favored (%)	98.24	97.74	98.08	98.76	98.77

Furthermore, the carboxylic acid moiety of **16a** not only formed a hydrogen bond with the main chain of Gly310 but also established a well-organized hydrogen bond network with Gly311, Val312, and Gly313 mediated by a water molecule. Moreover, the 1*H*-tetrazole moiety formed hydrogen bonds with the side chain of Ser83. Additionally, it may have interacted directly or through a water molecule with Arg278. Additional hydrophobic interactions occurred between the tail region of **16a** with Leu289, Pro309, and Pro314 of MTHFD2. It is noteworthy that compound **11**, lacking the tail region of **16a**, completely lost the inhibitory activity against MTHFD2, emphasizing the important role of the tail group in the compound’s inhibitory activity.

**Figure 3:**
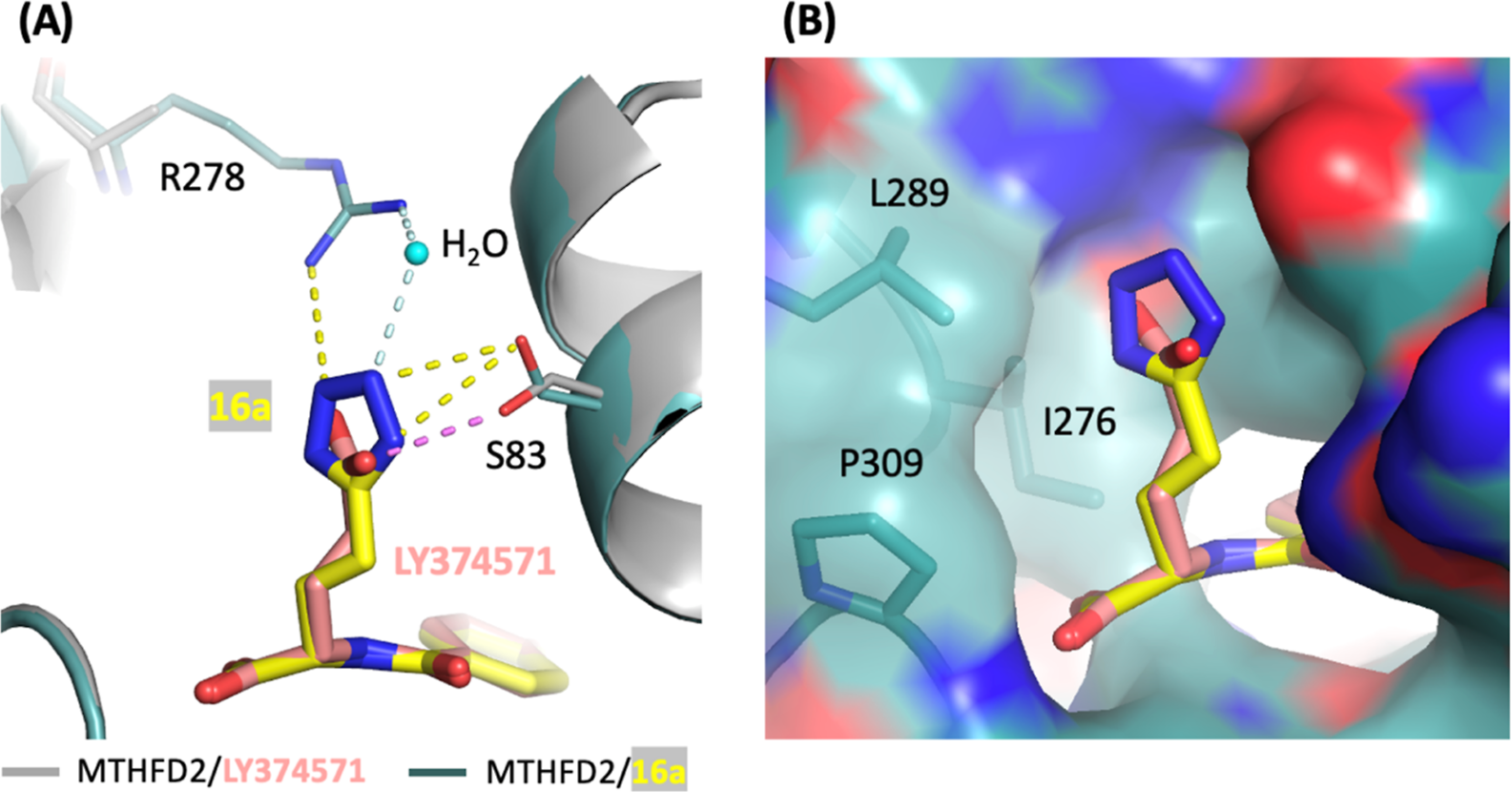
Superimposition of the crystal structure of MTHFD2/**16a** (PDB 9ISC, light teal/yellow) on MTHFD2/LY374571 (PDB 9IS9, gray/pink). For clarity, the NAD^+^ is omitted in the figure. (A) Interactions of the 1*H*-tetrazole moiety of **16a** with MTHFD2. Hydrogen-bond with protein and water -bridged interactions are shown in yellow and pale-cyan dashed lines, respectively. The water molecule is shown as a sphere (cyan). The hydrogen bond interaction of the γ-carboxylic acid moiety of LY374571 with MTHFD2 is shown in violet. The electron density map for the side chain of R278 is unclear; therefore, the side chain of R278 has been excluded from the MTHFD2/LY374571 structure. (B) The surface view around the tail part of **16a**.

When substituting the 1*H*-tetrazole moiety in compounds **16a** (and **16g**) with the γ-carboxylic acid of glutamic acid in LY374571 (and **16e**), it was found that **16a** and **16g** exhibited increased inhibitory potency against MTHFD2 compared to their counterparts with the γ-carboxylic acid substitution, LY374571 and **16e** (Table 1). Superimposition of the structures of MTHFD2/**16a** with MTHFD2/LY374571 revealed that the 1*H*-tetrazole moiety in **16a** exhibits an enhanced tendency for forming hydrogen bonds, either directly or through water bridging, with the side chain of Arg278 and Ser83 (Figure 3A). Moreover, the larger size of 1*H*-tetrazole moiety was found to form more extensive van der Waals contacts with neighboring residues such as Ile276, Leu289, and Pro309, as compared to its γ-carboxylic acid counterpart (Figure 3B). This observation suggests that the 1*H*-tetrazole moiety is better accommodated within the cavity, contributing to an enhanced overall structural fit. The collective impact of these interactions contributes to the increased potency of **16a** and **16g** against MTHFD2.

In comparison to **16a**, compound **16b** introduced an additional modification by replacing the carboxylic acid of **16a** with a 1*H*-tetrazole moiety. The considerable bulkiness of the 1*H*-tetrazole moiety in **16b** is likely to hinder the maintenance of an optimal distance required for forming hydrogen bonds with the main chain of Gly310. Moreover, the larger size of the 1*H*-tetrazole moiety in **16b** disrupted the water-mediated hydrogen bond network with residues Gly312, Val313, and Gly314. These structural alterations contribute to the decreased inhibitory activity of **16b** compared to **16a** against MTHFD2.

As revealed in both the structures of MTHFD2/**16a** and MTHFD2/LY374571, the amide moiety adjacent to the phenyl ring formed the hydrogen bond with the side chain of Asn87. The spatial configuration of this area is conducive to accommodating a small linker, such as the amide linker. When substituting it with a larger linker, exemplified by the sulfonyl moiety in compound **19**, steric hindrance interactions with surrounding residues, particularly Gly313, were introduced (Figure S2). The larger substitution also induced a shift in the α-carboxylic acid group, resulting in the loss of the hydrogen bond with the main chain of G310 and a water-mediated hydrogen bond network with Gly311, Val312, and Gly313 (Figure S2). Consequently, this alteration led to a significant perturbation in the interactions with MTHFD2, ultimately resulting in the complete loss of inhibitory activity against MTHFD2.

The substitution at the third position of phenyl ring exhibited a minor effect on the inhibition of MTHFD2. Comparative structural analysis of MTHFD2 in complex with compounds LY374571, **16d** and **16e** revealed similar interactions with the protein around this phenyl region, maintaining π–π interactions with Tyr84 and hydrophobic interactions with Tyr84, Ile276, Thr316, and Val317. Moreover, the replacement of the phenyl ring moiety (LY374571) with a pyridine group (**16h**) resulted in weaker π–π stacking interactions with the side chain of Tyr84, attributed to the perturbation of the π-electron cloud by the nitrogen atom within the pyridine ring. Consequently, this modification led to a reduction in activity against MTHFD2, suggesting the significance of the phenyl ring and its interactions with surrounding residues in modulating inhibitory activity.

As revealed in all cocrystal structures of MTHFD2 in complex with LY374571, **16a**, **16d**, **16e** and **16g**, the 2,5,6-triamino-3*H*-pyrimidin-4-one headgroup of these inhibitors was observed to form an extensive hydrogen bond network with MTHFD2 (Figure 2). The NH_2_ group at the sixth position of the triaminopyrimidine group was hydrogen bonded with the carboxy group of Leu133’s main chain. Additionally, it participated in a water-bridged hydrogen bond network with the side chains of Ser81 and Gln132, along with the main chain of Leu133. Modification of the NH_2_ group, either through its removal (**16j**) or substitution with a hydroxy group (**16i**), resulted in a significant decrease in inhibitory activity against MTHFD2. These results highlight the significant contribution of the NH_2_ group and its interaction network with surrounding residues to the compound’s potency.

Furthermore, the NH_2_ group at the second position of the triaminopyrimidine group formed hydrogen bonds with the main chain carboxyl group of Val131 and the side chain of Asp155. Removal of this NH_2_ group (**16l**) led to a drastic reduction in inhibitory activity against MTHFD2 by more than 100-fold. Moreover, introducing oxygen substitution to this NH_2_ group (**16k**) resulted in the complete loss of inhibitory activity toward MTHFD2. This substitution disrupted the strong hydrogen bond network formed with Val131 and Asp155, emphasizing the important role of this hydrogen bond network in the compound’s potency. The molecular modifications and structural biology study offer valuable insights into the intricate interplay of specific functional groups and their consequential influence on the interaction with MTHFD2.

### Elucidation of the Selectivity of Diaminopyrimidine-Based Derivatives by Structure Biology Studies

The analysis of compounds in Table 1 reveals distinctive selectivity patterns toward MTHFD2 compared to MTHFD1. Notably, among these compounds, **16d** and **16e** demonstrate higher selectivity with selectivity index (SI) values of 38.5 and 27.1, respectively.

To investigate the molecular basis of the selectivity toward MTHFD2 over MTHFD1, cocrystal structures of human MTHFD1 were determined in complex with **16a**, **16d**, **16e**, **16g**, and LY374571 in the presence of NADP^+^, with resolutions ranging from 1.99 to 2.50 Å (Table 3).

**Table 3:** X-ray Data and Structure Refinement for MTHFD1 Complex Structures

	MTHFD1/LY374571	MTHFD1/**16a**	MTHFD1/**16d**	MTHFD1/**16e**	MTHFD1/**16g**
resolution	27.48–1.99	26.21–2.28	29.89–2.09	29.69–2.06	30.00–2.50
space group	*C*222_1_	*C*222_1_	*C*222_1_	*C*222_1_	*C*222_1_
unit cell (α = β = γ = 90°) *a* (Å)	64.973	65.296	64.997	65.020	65.075
*b* (Å)	200.316	200.343	200.275	200.679	201.469
*c* (Å)	240.327	239.744	239.040	240.140	240.021
unique reflections	107841(10612)	72711 (7153)	93029 (9214)	97306 (9593)	55064 (5002)
*I*/σ	16.72 (2.03)	15.02 (2.02)	17.35 (2.05)	19.31 (2.02)	16.31 (2.22)
Rmerge (%)	6.4 (60.9)	8.5 (58.4)	6.3 (61.0)	5.7 (62.5)	10.2 (92.6)
completeness (%)	98.9 (99.6)	99.4 (99.6)	99.3 (100.0)	98.9 (100.0)	96.3 (92.5)
Rwork/Rfree	0.2006/0.2415	0.2368/0.2993	0.2110/0.2589	0.2064/0.2502	0.2128/0.2637
rmsd (bond) (Å)	0.016	0.010	0.008	0.008	0.009
rmsd (angle) (deg)	1.092	1.048	1.012	1.010	1.137
Ramachandran favored (%)	98.23	96.57	97.45	97.35	97.17

**Figure 4:**
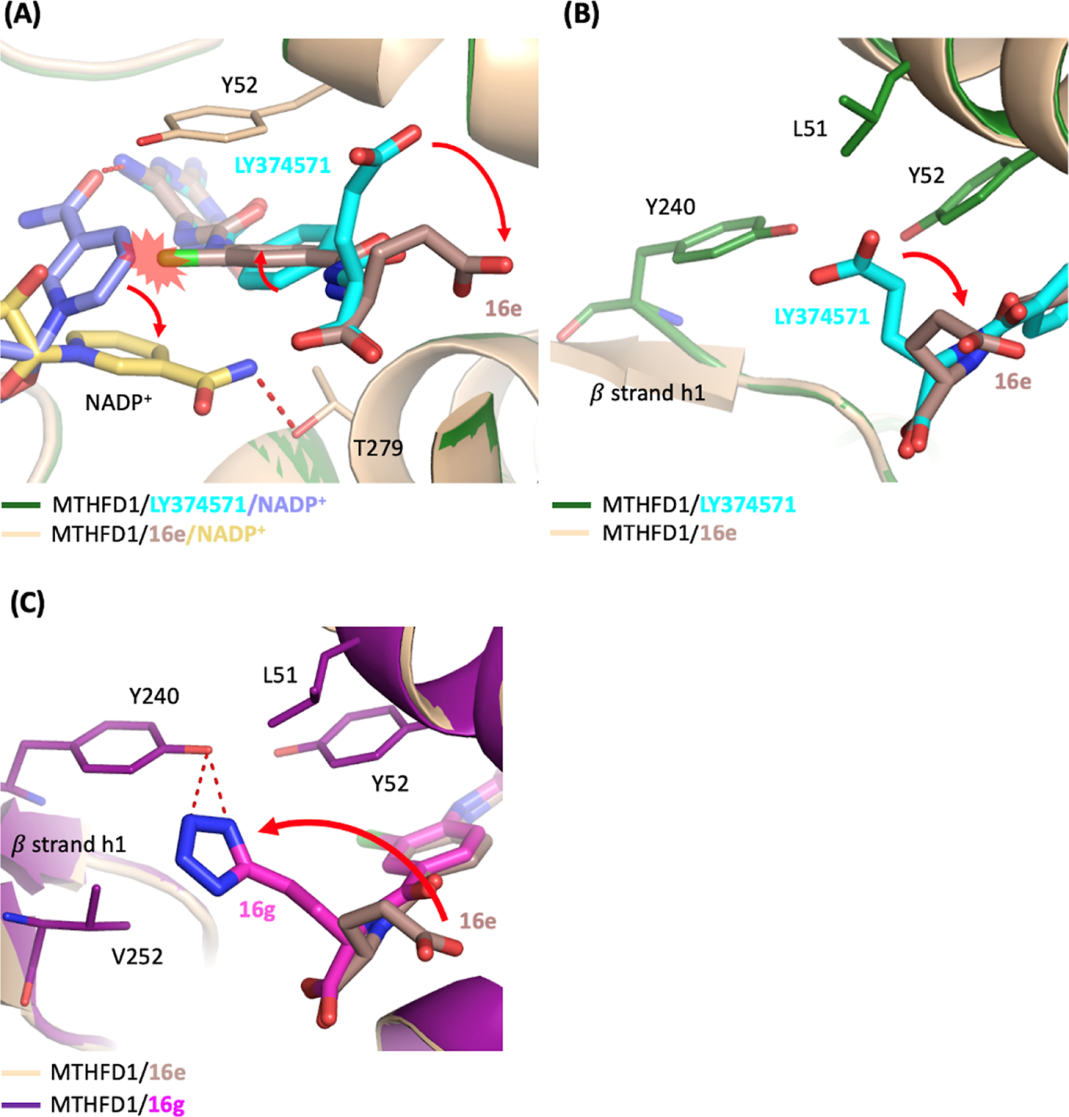
Superimposition of the crystal structure of MTHFD1/**16e**/NADP^+^ (PDB 9ISL, wheat/darksalmon/yellow-orange) on MTHFD1/LY374571/NADP^+^ (PDB 9ISE, forest/cyan/slate) or MTHFD1/**16g**/NADP^+^ (PDB 9ISR, deep purple/magenta). Hydrogen-bonds are shown in red dashed lines. (A) The presence of the chloride atom on the phenyl ring of compound **16e** induced the rotation of the nicotinamide group of NADP^+^. Additionally, the introduction of the chloride atom resulted in rotational adjustments of the phenyl ring and caused the γ-carboxylic acid to move away from β strand h1. (B) The shift of the γ-carboxylic acid in **16e** results in the loss or decrease of hydrophobic and van der Waals interactions with Leu51, Tyr52, and Tyr240. (C) The 1*H*-tetrazole moiety of **16g** shifted toward β strand h1 and was hydrogen bonded with Tyr240. For clarity, the NADP^+^ is omitted in the figure.

Superimposition of the MTHFD1/**16e** complex structure with MTHFD1/LY374571 complex structure uncovered several differences between them (Figure 4). First, in the structure of MTHFD1/LY374571, the nicotinamide group of NADP^+^ was hydrogen bonded with the NH_2_ moiety of the triaminopyrimidine headgroup of LY374571. However, the addition of a chloride substituent on the phenyl ring in **16e** induced a clash with the nicotinamide group of NADP^+^, leading to the shift of the nicotinamide group of NADP^+^ away from the triaminopyrimidine headgroup of **16e** and the consequent loss of the hydrogen bond between **16e** and NADP^+^ (Figure 4A) Instead, NADP^+^ in MTHFD1/**16e** structure was found to be hydrogen bonded with the side chain of Thr279. Second, in comparison with the MTHFD1/LY374571 structure, it was observed that the addition of the chloride atom in **16e** led to the rotation of the phenyl ring by approximately 26°, accompanied by the movement of the γ-carboxylic acid away from β strand h1 (Figure 4B). Moreover, the electron density of the γ-carboxylic acid in **16e** is weaker than that observed in LY374571. The shift of γ-carboxylic acid in **16e** resulted in the loss of the van der Waals interactions with Leu51, Tyr52 and Tyr240. These effects collectively contributed to the weaker inhibitory activity of **16e** toward MTHFD1 and consequently improved its selectivity to MTHFD2, as shown by the increase in the selectivity index from 7.9 to 27.1 compared with LY374571. Moreover, **16d**, which substituted the same position with a methyl group, similarly induced rotation of the phenyl ring and movement of the γ-carboxylic acid. Furthermore, as revealed in the structure of MTHFD1/**16d**, the density map of the nicotinamide group of NADP^+^ and the glutamic acid group appeared more unclear, indicating the flexibility and instability of NADP^+^ and the glutamic acid moiety of **16d**. This instability reflected on the much weaker inhibitory activity of **16d** against MTHFD1.

In contrast, compound **16a** and **16g**, featuring 1*H*-tetrazole moiety substitution at the γ-carboxylic acid of glutamic acid, exhibited potent inhibition against MTHFD1. In comparison with the structure of MTHFD1/**16e**, the structure of MTHFD1/**16g** revealed that the 1*H*-tetrazole moiety shifted toward β strand h1 and was hydrogen bonded with Tyr240 (Figure 4C). Moreover, the larger size of the 1*H*-tetrazole moiety relative to the carboxylic acid group, in conjunction with its interaction with Tyr240, promoted a movement toward β strand h1 and therefore formed van der Waals interactions with Val252 and Leu51. These structural changes facilitated an interaction network that increased the binding affinity of **16g** with MTHFD1. As a result, **16g** demonstrated potent inhibitory effects on MTHFD1, hence exhibiting reduced selectivity toward MTHFD2.

**Figure 5:**
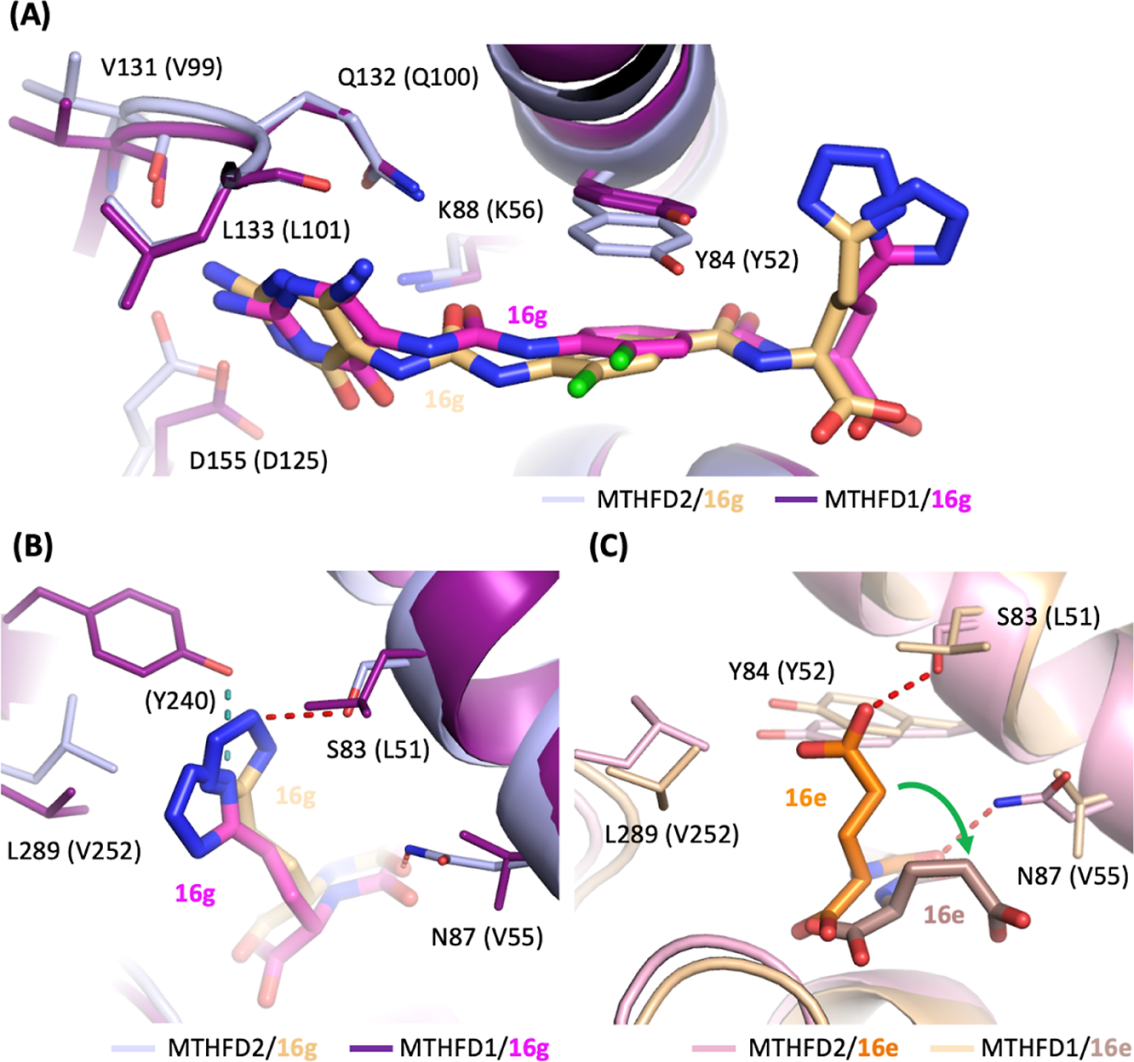
(A,B) Superimposition of the crystal structures of MTHFD2/**16g**/NAD^+^ (PDB 9IT6, light blue/light orange) with MTHFD1/**16g**/NADP^+^ (PDB 9ISR, deep purple/magenta) (C) Superimposition of the crystal structures of MTHFD2/**16e**/NAD^+^ (PDB 9IT3, light pink/orange) with MTHFD1/**16e**/NADP^+^ (PDB 9ISL, wheat/darksalmon). NAD^+^ or NADP^+^ is omitted for clarity. Hydrogen bonds between compound and MTHFD2 are shown as red dashed lines, while those with MTHFD1 are depicted as deep teal dashed lines. Residues in MTHFD1 are indicated in parentheses.

The superimposition of MTHFD1/**16g** and MTHFD2/**16g** revealed that the triaminopyrimidine headgroup, urea linker, and central phenyl ring of compound **16g** aligned well in both structures. The important interactions between the headgroup and urea linker of **16g** with residues Val131, Gln132, Leu133, Asp155, and Lys88 in MTHFD2 were retained in MTHFD1, where they corresponded to Val99, Gln100, Leu101, Asp125, and Lys56. Moreover, the π–π interactions between the central phenyl ring of **16g** and Tyr84 in MTHFD2 were similarly maintained in MTHFD1 with Tyr52 (Figure 5A). The 1H-tetrazole moiety of **16g** was positioned toward β strand h1 in both structures and interacted with the surrounding residues.

The differences between these two structures were attributed to the residue substitutions from Ser83 and Asn87 in MTHFD2 to Leu51 and Val55 in MTHFD1. These hydrophobic residue replacements in MTHFD1 prevented the formation of hydrogen bonds with the amide or 1H-tetrazole moiety of **16g**. Instead, a hydrogen bond was formed between the 1H-tetrazole moiety and Tyr240 in MTHFD1 (Figure 5B).

In comparison, the superimposition of MTHFD1/**16e** and MTHFD2/**16e** revealed notable differences in the tail region of the inhibitor. The γ-carboxylic acid of **16e** adopted different conformations in MTHFD1 and MTHFD2. In the MTHFD2/**16e** structure, the γ-carboxylic acid was positioned toward β strand h1, forming hydrogen bonds with Ser83 and hydrophobic interactions with Tyr84 and Leu289. However, in the MTHFD1/**16e** structure, the γ-carboxylic acid moiety shifted away, leading to the loss of these interactions (Figure 5C). This shift resulted in a weaker binding affinity of **16e** toward MTHFD1 compared to MTHFD2, as reflected by a higher selectivity of **16e** for MTHFD2 (SI = 27.1).

### Using LY374571 as A Reference Compound to Identify Optimal Cell Models for Evaluating MTHFD2 Inhibitors

**Figure 6:**
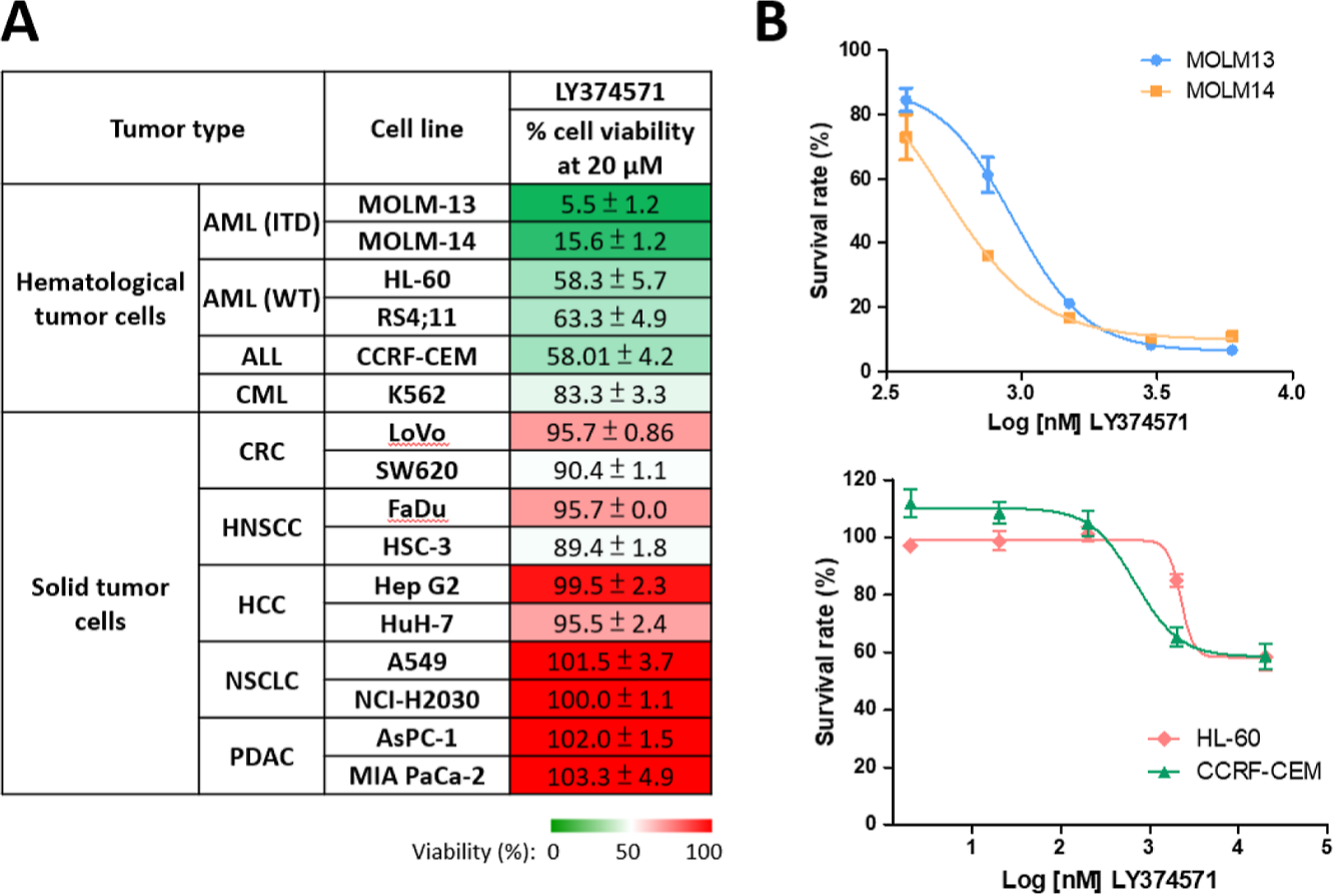
Using LY374571 as a reference compound to identify optimal cell models for evaluating MTHFD2 inhibitors. (A) The efficacy of LY374571 on cell growth inhibition in multiple hematological tumor cells and solid tumor cells. Cell viability was examined by MTT assay (hematological tumor cells) and methylene blue staining assay (solid tumor cells), respectively, after drug treatment for 72 h. In the experiment, the number of cells in the control group is set to 100%. The number of cells treated with 20 μM LY374571 is expressed as a percentage of cell viability relative to the control group. (B) Comparison of the efficacy of LY374571 on cell growth inhibition with increasing drug concentration for 72 h in MOLM-13 and MOLM-14 cells (upper panel) and HL-60 and CCRF-CEM (lower panel). Data was from three independent experiments and shown as means ± SD.

Due to promising enzymatic activity demonstrated by diaminopyrimidine-based analogues, further analysis is required to evaluate their potential in inhibiting cancer cell growth. In order to identify the optimal cell model for assessing MTHFD2 inhibitors, LY374571 served as a reference compound to evaluate its inhibitory effect on cell growth across a panel of hematological and solid tumor cells. After 72 h of treatment with 20 μM LY374571, hematological tumor cells demonstrated greater growth inhibition efficacy compared to solid tumor cells (Figure 6A). Subsequently, the four most sensitive cell lines- MOLM-13, MOLM-14, HL-60, and CCRF-CEM cells were selected for investigating the growth inhibitory effect of LY374571. Among them, MOLM-13 and MOLM-14, both acute myeloid leukemia (AML) cells with FLT3 internal tandem duplication (FLT3-ITD) mutations,^14,34^ exhibited heightened sensitivity to LY374571 compared to HL-60 (an AML cell line with wild type FLT3) and CCRF-CEM (an acute lymphoblastic leukemia cell line) (Figure 6B).

FLT3, a member of the class III receptor tyrosine kinase family, is abundantly expressed in hematopoietic cells^35^ and plays a key role in cell survival, proliferation, and differentiation.^36^ In acute myeloid leukemia (AML) patients, FLT3-ITD mutations result in a gain-of-function of the receptor.^37^ While the exact mechanism underlying the heightened sensitivity of FLT3-ITD AML cells to MTHFD2 inhibitors remains unclear, one possible explanation is the significantly increased production of reactive oxygen species (ROS) in FLT3-ITD AML cells,^38,39^ which raises the demand for NAD(P)H to support antioxidant defenses. One-carbon metabolism contributes approximately 40% of NAD(P)H production,^40^ and MTHFD2 plays a key role in maintaining redox homeostasis.^41,42^ Furthermore, FLT3-ITD-driven leukemias rely on serine for both proliferation and survival, upregulating serine biosynthesis and one-carbon metabolism enzymes, including MTHFD2.^43^ This increased need for antioxidant activity and dependence on serine metabolism likely enhances the sensitivity of FLT3-ITD AML cells to MTHFD2 inhibition.

Furthermore, the MOLM-14 cell line showed greater sensitivity to LY374571-mediated growth inhibition than MOLM-13, with respective GI_50_ values of 630 ± 30 and 830 ± 130 nM. Therefore, MOLM-14 was selected as the optimal model for subsequent investigations based on its pronounced sensitivity to LY374571.

In the study by Pikman et al., an analysis of gene expression databases from AML patients revealed that genes related to the one-carbon pathway were highly expressed, particularly in the AML FLT3-ITD mutation group.^14^ The authors explored MTHFD2 as a potential therapeutic target for AML using MTHFD2 knockdown approaches; however, they did not validate their findings with small-molecule MTHFD2 inhibitors. Therefore, combining the insights from this previous study^14^ with the results of our research provides robust information for optimizing the cell screening platform for MTHFD2 inhibitors.

### The Degree of Inhibition of MTHFD1 and MTHFD2 Enzymatic Activities by the Compounds Correlates with Their Ability to Inhibit MOLM-14 Cell Growth

The compounds **16a** and **16g** demonstrate relatively strong inhibition of both MTHFD1 and MTHFD2 (Table 1) and significantly inhibit MOLM-14 cell growth (Table 4). The extent of their inhibition against MTHFD1 and MTHFD2 enzymatic activities correlates with their efficacy in inhibiting MOLM-14 cell growth (Table 4). Both MTHFD1 and MTHFD2 play pivotal roles in one-carbon metabolism, but their distinct expression profiles may lead to significantly different outcomes upon inhibition.^1^ MTHFD1 functions as a housekeeping gene and is widely expressed across various tissues.^1,44^ MTHFD1 is a critical enzyme in normal cells for maintaining normal cellular metabolism, supporting functions such as cell division, growth, and repair.^21,44^ In contrast, MTHFD2 is predominantly expressed in embryonic and transformed cells, with minimal or absent expression in most adult differentiated tissues.^4,45^

**Table 4:** Analysis of the Ability of Diaminopyrimidine Derivatives to Inhibit the Growth of MOLM-14 Cells and Comparison with IC_50_ and Selectivity Index of MTHFD1/2

ID	MOLM-14, GI_50_ (nM)	enzyme-based assay		
		MTHFD1 IC_50_ (nM)	MTHFD2 IC_50_ (nM)	SI
LY374571	630 ± 30	630 ± 60	80 ± 10	7.9
**16a**	20 ± 10	120 ± 0	22 ± 4	5.5
**16b**	810 ± 110	380 ± 10	150 ± 20	2.5
**16c**	590 ± 110	790 ± 60	910 ± 110	0.87
**16d**	893 ± 8	5160 ± 1170	134 ± 30	38.5
**16e**	720 ± 20	1790 ± 100	66 ± 13	27.1
**16f**	780 ± 210	590 ± 30	90 ± 5	6.6
**16g**	120 ± 60	230 ± 0	40 ± 10	5.8
**16h**	5740 ± 930	4140 ± 200	470 ± 30	8.8
**16j**	>20000	2200 ± 760	530 ± 110	4.2
**16l**	5490 ± 770	>10000	8330 ± 2190	

The critical role of MTHFD1 in cellular function is evident from studies showing that homozygous knockout mutations in *Mthfd1* result in embryonic lethality in mice.^46,47^ Additionally, several reports link MTHFD1 deficiency to severe combined immunodeficiency (SCID) in patients.^48−52^ Along with the variable phenotypes of SCID, MTHFD1 deficiency presents a spectrum of clinical conditions, including megaloblastic anemia, atypical hemolytic uremic syndrome, anisocytosis, autoimmune diseases, and microangiopathy. Notably, patients with pathogenic MTHFD1 variants exhibited MTHFD1 protein levels at approximately 5–50% of controls, and the enzyme’s dehydrogenase activity was nearly undetectable.^53^

Given these findings, compounds targeting MTHFD1 may pose substantial risks for adverse effects. In contrast, compounds with higher specificity for MTHFD2 offer the potential for a broader therapeutic window, minimizing toxicity and reducing the likelihood of side effects. Therefore, compounds with high selectivity for MTHFD2, such as **16d** and **16e**, emerge as more promising compounds for advancement (Table 1). These compounds demonstrate significant selectivity for MTHFD2 and despite their moderate inhibition of cell growth (Table 4), they show potential as therapeutic agents with potentially reduced side effects. Furthermore, comparing the inhibitory activity of MTHFD2 and cell growth inhibition, **16e** is superior to **16d**. Compound **16e** has a lower IC_50_ value against MTHFD2 (Table 1) and demonstrated cell growth inhibition in AML MOLM-14 cells (Table 4 and Figure S3), while showing no effect on normal cells (Figure S3), these finding making it a more promising compound for further pharmacokinetic studies and in vivo efficacy assessments.

### Compounds **16a** and **16e** Produce Synergistic Effects in MOLM-14 Cells in Combination with Alimta

**Figure 7:**
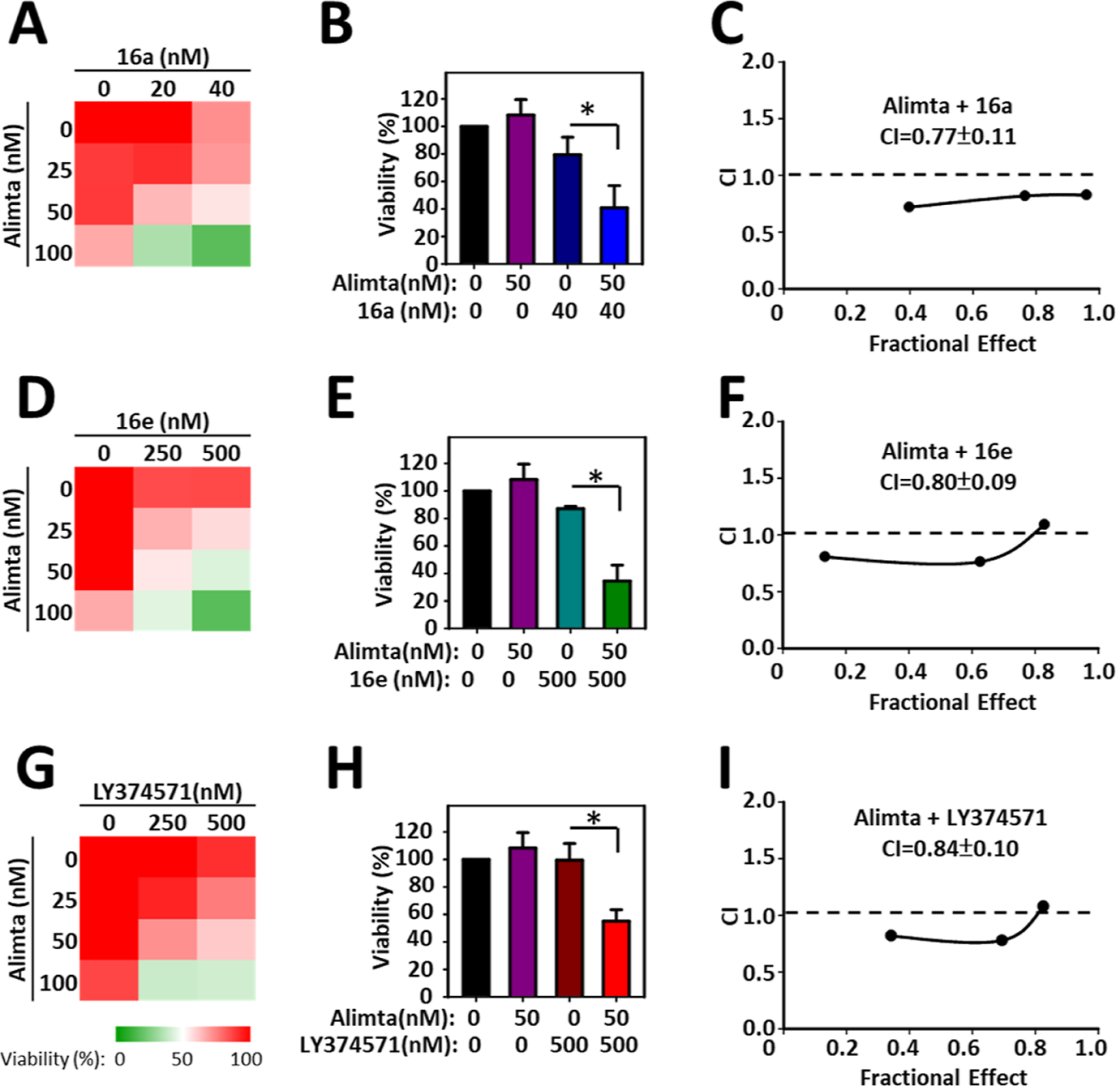
Investigation of combination benefits of Alimta with MTHFD2 inhibitors in MOLM-14 cells. MOLM-14 cells were treated with Alimta and MTHFD2 inhibitors (**16a**, **16e**, or LY374571) alone or in combination for 72 h. Cell viability was evaluated by MTT assay and displayed by heatmap (A,D,G). Cell viability is expressed as a percentage relative to the control group, with red indicating higher viability and green indicating lower viability. Bar graphs illustrate cell viability after treatment with Alimta and compound **16a** (B), **16e** (E), or LY374571 (H). The results are presented as mean ± standard deviation (SD), and statistical significance is indicated by an asterisk (*), *p* < 0.05. combination index (CI) plot for Alimta with compound **16a** (C), **16e** (F), or LY374571 (I). The CI values were calculated by CalCusyn software to evaluate the synergistic effect of the two compounds. A CI value less than 1 indicates synergy, a CI value equal to 1 indicates an additive effect, and a CI value greater than 1 indicates antagonism.

Alimta (Pemetrexed) is a clinically used antifolate chemotherapy drug that targets several key enzymes involved in one-carbon metabolism, including thymidylate synthase (TS), dihydrofolate reductase (DHFR), and glycinamide ribonucleotide formyltransferase (GARFT).^54^ One-carbon metabolism is crucial for the synthesis of purines and thymidine, which are necessary for DNA replication and repair. We hypothesized that a more comprehensive inhibition of one-carbon metabolism could produce a stronger synergistic anticancer effect. Therefore, the combined effects of the newly developed MTHFD2 inhibitors **16a** and **16e** with Alimta were investigated in combination experiments. Heatmaps illustrate the cell viability percentages of MOLM-14 cells treated with increasing concentrations of Alimta alongside compound **16a** (Figure 7A), compound **16e** (Figure 7D), or reference compound LY374571 (Figure 7G). All combinations significantly reduced cell viability compared to individual treatments (Figure 7B,E,H). Combination Index (CI) plots further revealed the interaction between Alimta and **16a**, **16e**, or LY374571. A CI value less than 1 indicates synergistic effect. The CI between Alimta and **16a** is 0.77 ± 0.11 (Figure 7C), between Alimta and **16e** is 0.80 ± 0.09 (Figure 7F), and between Alimta and LY374571 is 0.84 ± 0.10 (Figure 7I), suggesting a synergistic interaction in all cases.

### Pharmacokinetic Profiles of **16e** and LY374571 Following Intravenous Administration in Mice

Due to its potent enzymatic inhibition, high selectivity for MTHFD2, and effective cell growth inhibition, compound **16e** emerges as a promising compound for further pharmacokinetic investigation. LY374571 was included in the study for comparative analysis. The pharmacokinetic parameters of LY374571 and **16e** were assessed following a single intravenous dose of 2 mg/kg. LY374571 exhibited a half-life (*T*_1/2_) of 1.6 h, a clearance (CL) of 70.7 mL/min/kg, a volume of distribution at steady state (*V*_ss_) of 14.7 L/kg and the AUC_0-inf_ of 542 ng/mL × h. In comparison, compound **16e** demonstrated a longer half-life (*T*_1/2_) of 6.5 h, a much lower CL of 12.3 mL/min/kg, and a significantly smaller volume of distribution at steady state (*V*_ss_) of 0.6 L/kg. The AUC_0-inf_ was notably higher at 2702 ng/mL × h. These results suggest that **16e** has a longer systemic exposure and lower clearance compared to LY374571, indicating its prolonged presence in the body and slower elimination (Table 5). Therefore, compound **16e** exhibits a more favorable pharmacokinetic profile.

**Table 5:** Pharmacokinetic Profiles of LY374571 and **16e**

compound	IV (dose: 2 mg/kg)			
	*T*_1/2_ (hr)	CL (mL/min/kg)	*V*_ss_ (l/kg)	AUC_(0-inf)_ (ng/mL× h)
LY374571	1.6	70.7	14.7	542
**16e**	6.5	12.3	0.6	2702

### In Vivo Anticancer Efficacy of **16e** and LY374571 in MOLM-14 Xenograft Tumor Model

**Figure 8:**
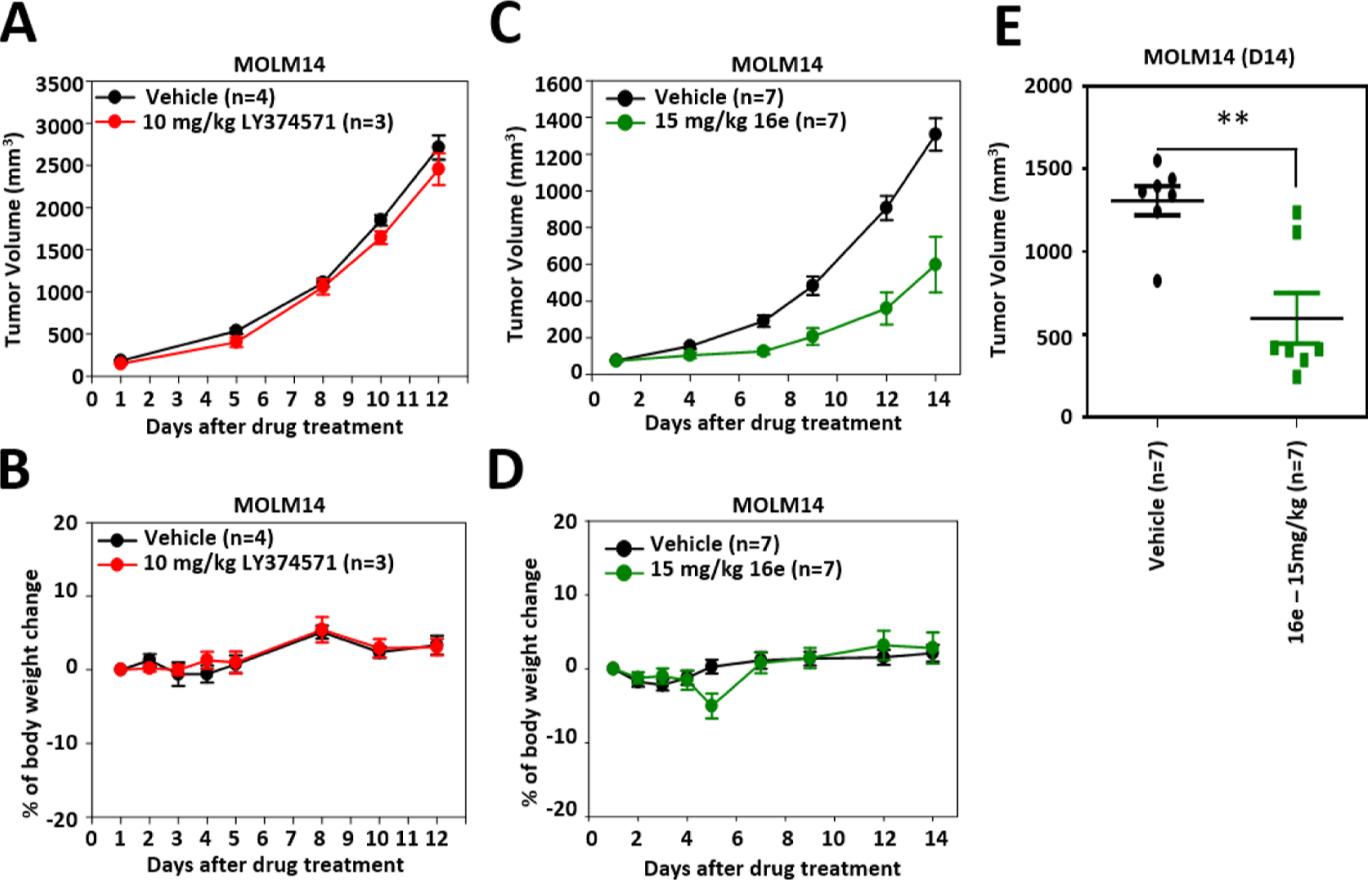
In vivo antitumor efficacy of MTHFD2 inhibitors in MOLM-14 xenograft mouse model. MOLM-14 cells were subcutaneously implanted into NOD SCID mice and the tumor-bearing mice were treated with LY374571 (10 mg/kg, intravenously injected, QD, 2 cycles of 5-on/2-off). Tumor volume (A) and body weight (B) were measured twice weekly. MOLM-14 cells were subcutaneously implanted into NOD SCID mice and the tumor-bearing mice were treated with the compound **16e** (15 mg/kg, intravenously injected, QD). Tumor volume (C) and body weight (D) were measured twice weekly. (E) Tumor volumes of mice treated with vehicle or compound **16e** on day 14 after drug administration. Each point represents the tumor volume of an individual mouse (*n* = 7 per group). The data are presented as mean ± standard deviation (SD). Statistical significance between the vehicle-treated group and the **16e**-treated group is indicated by double asterisks (**), *p* < 0.01.

In this study, the effects of LY374571 and **16e** on tumor growth and body weight were investigated using the MOLM-14 xenograft model. MOLM14-bearing mice were treated with 10 mg/kg LY374571 and 15 mg/kg **16e**, respectively. These doses were selected based on the maximum solubility of each compound in the intravenous (*i.v.*) vehicle solution. LY374571 exhibited minimal antitumor activity, with a tumor growth inhibition (TGI) of 8.8% on day 12 (Figure 8A). Notably, the anticancer efficacy of **16e** was significantly effective from day 7 (TGI 75.7%) to day 14 (TGI 57.4%) (Figure 8C). Day 14 after drug administration, the vehicle-treated group (*n* = 7) exhibited a mean tumor volume of approximately 1307 mm^3^, with individual tumor volumes ranging from around 825 to 1550 mm^3^. In contrast, the group treated with 15 mg/kg of **16e** (*n* = 7) showed a significantly lower mean tumor volume of about 598 mm^3^, with individual tumor volumes ranging from approximately 245 to 1237 mm^3^ (Figure 8E). Notably, neither LY374571 nor **16e** treatment resulted in significant change in the body weight of the mice throughout the study period (Figure 8B,D). These results highlight the substantial effect of compound **16e** in reducing tumor growth, further supporting its potential as a therapeutic agent in cancer treatment.

## Conclusions

In this study, selective 2,4-diamino-6-oxo-1,6-dihydropyrimidin-5-yl ureido based MTHFD2 inhibitors were identified through systematic chemical structural modification and SAR studies. The lead optimization strategy outlined in Table 1 demonstrates several key findings: (1) glutamic acid is preferred as the terminal amino acid moiety, (2) the γ-carboxyl group of glutamic acid could be substituted with an acid bioisostere such as 1*H*-tetrazole, (3) the amide moiety at the 1-position and the urea linker at the 4-position of the central phenyl ring are important functional groups, and (4) modifications of the diaminopyrimidine headgroup result in a significant loss of potency. Furthermore, structural biology investigations provide detailed insights into the molecular mechanisms governing their specificity toward MTHFD2. Comparative analysis of the structures of MTHFD2 complexed with 2,4-diaminopyrimidine-based derivatives and those of MTHFD1 revealed that additional substituents on the phenyl ring of inhibitors significantly altered binding interactions, including a shift in the nicotinamide group of NADP^+^ and disruption of important hydrogen bonds with cofactors in MTHFD1. Furthermore, these substituents induced conformational changes in the phenyl ring and γ-carboxylic acid, resulting in weaker inhibitory activity against MTHFD1 and enhanced selectivity for MTHFD2. Conversely, substitutions with larger moieties in the tail group prompted movements toward β strand h1 to form interactions with MTHFD1, leading to increased inhibitory activity against MTHFD1 and reduced selectivity for MTHFD2. In conclusion, our study reveals the important role of substitutions in the phenyl ring and tail group in modulating the potency and selectivity toward MTHFD2 within the diaminopyrimidine series. These findings highlight the potential for achieving enhanced potency and selectivity toward MTHFD2 through chemical modifications in these specific structural moieties.

Moreover, this study pioneered the establishment of a comprehensive cell screening platform for MTHFD2 inhibitors using a diverse panel of leukemia and solid tumor cell lines. Notably, acute myeloid leukemia (AML) cells harboring FLT3-ITD mutations exhibited particularly favorable responses to these inhibitors. Additionally, synergistic effects were observed when potential compounds were combined with Alimta. Through a combination of medicinal chemistry, structural biology, and comprehensive in vitro and in vivo bioactivity assays, compound **16e** emerged as a promising candidate, demonstrating superior inhibitory activity and specificity for MTHFD2. Compound **16e** also showed favorable pharmacokinetic profiles and exhibited potent antitumor efficacy in MOLM-14 xenograft models. These findings not only contribute to the understanding of MTHFD2-specific inhibition mechanisms but also underscore the potential therapeutic implications for cancers treatment by targeting MTHFD2.

## Experiment Section

### Chemistry Methods

All commercial chemicals and solvents are reagent grade and were used without further treatment unless otherwise noted. ^1^H NMR spectra were obtained with a Varian Mercury-300, a Varian Mercury-400 or a Bruker Advance Neo 600 spectrometer. Chemical shifts were recorded in parts per million (ppm, δ) and were reported relative to the solvent peak or TMS. LC/MS data were measured on an Agilent MSD-1100 ESI-MS/MS System. High-resolution mass spectra (HRMS) were measured with a Thermo Finnigan (TSQ Quantum) electrospray ionization (ESI) mass spectrometer. Flash column chromatography was done using silica gel (Merck Kieselgel 60, no. 9385, 230–400 mesh ASTM). Reactions were monitored by TLC using Merck 60 F_254_ silica gel glass backed plates (5 × 10 cm); zones were detected visually under ultraviolet irradiation (254 nm) or by spraying with phosphomolybdic acid reagent (Aldrich) followed by heating at 80 °C. All starting materials and amines were commercially available unless otherwise indicated. The purity of compounds was determined by a Hitachi 2000 series HPLC system and a Waters Acquity UPLC/BSM with PhotoDiode Array detector. Purity of all target compounds shown in Table 1 was over 95% based on a reverse phase C18 column (Agilent ZORBAX Eclipse XDB-C18 5 μm, 4.6 mm × 150 mm) under the following gradient elution conditions: Mobile phase A-acetonitrile (10 to 90%, 0 to 45 min) and mobile phase B-2 mM NH_4_OAc aqueous solution containing 0.1% formic acid (90 to 10%, 0 to 60 min). The flow-rate was 0.5 mL/min and the injection volume was 20 μL. The system operated at 25 °C. Peaks were detected at λ = 254 nm.

### General Procedure for the Preparation of Compounds **11** and **13**

Targeted compound **11** and intermediate **13** were synthesized according to the literature report.^31^ Representative compounds **11** and **13i** (a key intermediate to **16i**) are selected to show their NMR and mass data.

### 1-(2,4-Diamino-6-oxo-1,6-dihydropyrimidin-5-yl)-3-(4-nitrophenyl)urea (**11**)

^1^H NMR (400 MHz, DMSO-*d*_6_): δ 9.98 (s, 1H), 9.28 (s, 1H), 8.14 (d, *J* = 9.2 Hz, 2H), 7.68 (d, *J* = 8.8 Hz, 2H), 6.87 (s, 1H), 6.16 (s, 2H), 5.91 (s, 2H). MS (ES^+^) *m*/*z*: calcd for C_11_H_11_N_7_O_4_, 305.1, found, 306.1 (M + H)^+^.

### 4-{[(2-Amino-4-hydroxy-6-oxo-1,6-dihydropyrimidin-5-yl)carbamoyl]amino}benzoic Acid (**13i**)

1H NMR (400 MHz, DMSO-*d*_6_): δ 11.31 (s, 2H), 8.91 (s, 1H), 7.81 (d, *J* = 8.8 Hz, 2H), 7.52 (d, *J* = 8.7 Hz, 2H), 6.80 (s, 1H), 6.67 (s, 2H); MS (ES^+^) *m*/*z*: calcd for C_12_H_11_N_5_O_5_, 305.1; found, 306.1 (M + H)^+^.

Targeted compounds **16**, **19** and **23** were prepared following the previous publication with some modifications.^24,31^ To a solution of **13** (1.0 equiv) in dry DMSO at room temperature was added HOSu (2 equiv) and EDC HCl (2 equiv) and the resultant mixture was stirred at room temperature for 3–4 h. The reaction mixture was added amine **14** or **15** (4.0 equiv). After stirring at room temperature overnight, the solvent DMSO were removed under reduced pressure and the residue was washed with water and MeOH to yield intermediate ester. The ester (1.0 equiv) was suspended in water and added 1 M NaOH solution (2–4 equiv). The reaction was stirred at room temperature for 1–2 h. 1 M HCl was added until pH ∼ 2 and the product was collected by filtration. The crude product was washed with water and MeOH. The resultant solid was collected by filtration and dried in vacuo or was further purified by SiliaBondR C18 (17%) eluted with 0–20% MeOH in water containing 0.1% formic acid or trifluoroacetic acid to yield desired diacid or monoacid **16**.

### (2*S*)-2-[(4-{[(2,4-Diamino-6-oxo-1,6-dihydropyrimidin-5-yl)carbamoyl]amino}benzoyl)amino]-4-(1*H*-tetrazol-5-yl)butanoic Acid (**16a**)

^1^H NMR (400 MHz, DMSO-*d*_6_): δ 10.00 (s, 1H), 8.83 (s, 1H), 8.52 (d, *J* = 7.6 Hz, 1H), 7.80 (d, *J* = 8.8 Hz, 2H), 7.52 (d, *J* = 8.8 Hz, 2H), 6.73 (s, 1H), 6.16 (s, 2H), 5.88 (s, 2H), 4.43–4.38 (m, 1H), 2.98 (t, *J* = 7.7 Hz, 2H), 2.33–2.29 (m, 1H), 2.23–2.14 (m, 1H); ^13^C NMR (150 MHz, DMSO-*d*_6_): δ 173.50, 166.21, 161.75, 160.30, 155.93, 154.73, 153.34, 143.65, 128. 37, 126.10, 116.57, 89.36, 52.07, 28.54, 20.31; MS (ES^+^) *m*/*z*: calcd for C_17_H_20_N_11_O_5_, 458.2; found, 458.1 (M + H)^+^; HRMS (ESI^–^): calcd for C_17_H_18_N_11_O_5_, 456.1492; found, 456.1492 (M – H)^−^; UPLC *t*_R_ = 6.82 min, 97.5%.

### 4-{[(2,4-Diamino-6-oxo-1,6-dihydropyrimidin-5-yl)carbamoyl]amino}-*N*-[(1*S*)-1,3-di(1*H*-tetrazol-5-yl)propyl]benzamide (**16b**)

^1^H NMR (400 MHz, DMSO-*d*_6_): δ 9.97 (br s, 1H), 8.86 (d, *J* = 8.0 Hz,, 1H), 8.85 (br s, 1H), 7.82 (d, *J* = 8.0 Hz,, 2H), 7.53 (d, *J* = 8.0 Hz, 2H), 6.71 (s, 1H), 6.14 (s, 2H), 5.87 (s, 2H), 5.49–5.43 (m, 1H), 3.05–3.01 (m, 2H), 2.60–2.40 (m, 2H); ^13^C NMR (100 MHz, DMSO-*d*_6_): δ 166.02, 161.69, 160.30, 158.27, 155.56, 154.70, 153.32, 143.74, 128.52, 125.89, 116.62, 89.36, 44.19, 30.60, 19.81; MS (ES^+^) *m*/*z*: calcd for C_17_H_20_N_15_O_3_, 482.2; found, 482.4 (M + H)^+^; HRMS (ESI^–^): calcd for C_17_H_18_N_15_O_3_, 480.1717; found, 480.1714 (M – H)^−^; UPLC *t*_R_ = 5.02 min, 96.9%.

### *N*-2-(4-{[(2,4-Diamino-6-oxo-1,6-dihydropyrimidin-5-yl)carbamoyl]amino}benzoyl)-l-glutamine (**16c**)

^1^H NMR (400 MHz, DMSO-*d*_6_): δ 8.84 (br s, 1H), 8.48 (d, *J* = 6.8 Hz, 1H), 7.80 (d, *J* = 8.8 Hz, 2H), 7.52 (d, *J* = 8.4 Hz, 2H), 7.32 (s, 1H), 6.82 (br s, 4H), 6.33 (br s, 1H), 4.32 (br s, 1H), 2.21 (m, 2H), 2.07 (m, 1H), 1.94 (m, 1H); ^13^C NMR (150 MHz, DMSO-*d*_6_): δ 173.89, 173.80, 166.09, 161.71, 160.28, 154.74, 153.34, 143.59, 128.32, 126.18, 116.66, 89.37, 52.41, 31.63, 26.26; MS (ES^+^) *m*/*z*: calcd for C_17_H_21_N_8_O_6_, 433.2, found, 433.1 (M + H)^+^; HRMS (ESI^–^): calcd for C_17_H_19_N_8_O_6_, 431.1428; found, 431.1429 (M – H)^−^; UPLC t_R_ = 6.71 min, 95.0%.

### *N*-(4-{[(2,4-Diamino-6-oxo-1,6-dihydropyrimidin-5-yl)carbamoyl]amino}-3-methylbenzoyl)-l-glutamic Acid (**16d**)

^1^H NMR (600 MHz, DMSO-*d*_6_): δ 12.39 (br s, 2H), 8.39 (d, *J* = 6.6 Hz, 1H), 8.02 (br d, *J* = 25.2 Hz, 2H), 7.70 (s, 1H), 7.66 (d, *J* = 7.8 Hz, 1H), 7.38 (s, 1H), 6.91 (s, 2H), 6.42 (s, 2H), 4.38 (s, 1H), 2.34 (s, 2H), 2.26 (s, 3H), 2.08 (br s, 1H), 1.94 (br s, 1H); ^13^C NMR (150 MHz, DMSO-*d*_6_): δ 174.03, 173.73, 166.43, 161.18, 160.38, 154.71, 153.22, 141.51, 129.57, 126.38, 125.84, 125.01, 118.19, 89.48, 51.92, 30.51, 26.02, 17.93; MS (ES^+^) *m*/*z*: calcd for C_18_H_22_N_7_O_7_, 448.2; found, 448.1 (M + H)^+^; HRMS (ESI^–^): calcd for C_18_H_20_N_7_O_7_, 446.1424; found, 446.1427 (M – H)^−^; UPLC *t*_R_ = 4.62 min, 96.4%.

### *N*-(3-Chloro-4-{[(2,4-diamino-6-oxo-1,6-dihydropyrimidin-5-yl)carbamoyl]amino}benzoyl)-l-glutamic Acid (**16e**)

^1^H NMR (400 MHz, DMSO-*d*_6_): δ 12.38 (br s, 2H), 10.02 (br s, 1H), 8.54 (d, *J* = 8.0 Hz, 1H), 8.36 (d, *J* = 8.0 Hz, 1H), 8.30 (br s, 1H), 7.97 (d, *J* = 4.0 Hz, 1H), 7.78 (dd, *J* = 8.0, 4.0 Hz, 1H), 7.68 (br s, 1H), 6.18 (br s, 2H), 5.97 (br s, 2H), 4.42–4.30 (m, 1H), 2.34 (t, *J* = 8.0 Hz, 2H), 2.14–2.00 (m, 1H), 2.00–1.86 (m, 1H); ^13^C NMR (150 MHz, DMSO-*d*_6_): δ 173.88, 173.46, 164.96, 161.55, 160.18, 154.15, 153.38, 139.65, 128.28, 127.09, 126.99, 119.86, 118.60, 88.90, 51.99, 30.43, 25.92; MS (ES^+^) *m*/*z*: calcd for C_17_H_19_ClN_7_O_7_, 468.1; found, 468.1 (M + H)^+^; HRMS (ESI^–^): calcd for C_17_H_17_ClN_7_O_7_, 466.0878; found, 466.0878 (M – H)^−^; UPLC *t*_R_ = 5.90 min, 96.3%.

### *N*-(4-{[(2,4-Diamino-6-oxo-1,6-dihydropyrimidin-5-yl)carbamoyl]amino}-3-fluorobenzoyl)-l-glutamic Acid (**16f**)

^1^H NMR (400 MHz, DMSO-*d*_6_): δ 8.77 (s, 1H), 8.53 (d, *J* = 4.0 Hz,, 1H), 8.26 (t, *J* = 8.0 Hz,, 1H), 7.74 (d, *J* = 8.0 Hz, 1H), 7.69 (d, *J* = 4.0 Hz, 1H), 7.40 (s, 1H), 6.95 (br s, 1H), 6.45 (br s, 1H), 4.41–4.32 (m, 1H), 2.35 (t, *J* = 4.0 Hz, 2H), 2.13–2.03 (m, 1H), 1.99–1.89 (m, 1H); ^13^C NMR (150 MHz, DMSO-*d*_6_): δ 173.91, 173.48, 165.12, 159.99, 154.13, 152.52, 151.42, 149.82, 131.51 (d, *J* = 10.5 Hz), 126.56, 124.07, 118.58, 114.00 (d, *J* = 21 Hz), 88.95, 52.02, 30.45, 25.93; MS (ES^+^) *m*/*z*: calcd for C_17_H_19_FN_7_O_7_, 452.1, found, 452.4 (M + H)^+^; HRMS (ESI^–^): calcd for C_17_H_17_FN_7_O_7_, 450.1174; found, 450.1175 (M – H)^−^; UPLC *t*_R_ = 5.13 min, 98.2%.

### (2*S*)-2-[(3-Chloro-4-{[(2,4-diamino-6-oxo-1,6-dihydropyrimidin-5-yl)carbamoyl]amino}benzoyl)amino]-4-(1*H*-tetrazol-5-yl)butanoic Acid (**16g**)

^1^H NMR (400 MHz, DMSO-*d*_6_): δ 10.03 (br s, 1H), 8.68 (d, *J* = 4.0 Hz, 1H), 8.38 (d, *J* = 8.0 Hz, 1H), 8.31 (br s, 1H), 7.98 (d, *J* = 4.0 Hz, 1H), 7.79 (dd, *J* = 8.0, 4.0 Hz, 1H), 7.69 (br s, 1H), 6.19 (br s, 2H), 5.99 (br s, 2H), 4.43–4.38 (m, 1H), 3.00 (t, *J* = 8.0 Hz, 2H), 2.35–2.30 (m, 1H), 2.23–2.16 (m, 1H); ^13^C NMR (150 MHz, DMSO-*d*_6_): δ 173.31, 164.94, 161.76, 160.20, 156.20, 154.16, 153.44, 139.64, 128.29, 127.06, 119.89, 118.62, 88.89, 52.28, 28.59, 20.44; MS (ES^+^) *m*/*z*: calcd for C_17_H_19_ClN_11_O_5_, 492.1, found, 492.2 (M + H)^+^; HRMS (ESI^–^): calcd for C_17_H_17_ClN_11_O_5_, 490.1103; found, 490.1102 (M – H)^−^; UPLC *t*_R_ = 6.14 min, 96.9%.

### *N*-[(6-{[(2,4-Diamino-6-oxo-1,6-dihydropyrimidin-5-yl)carbamoyl]amino}pyridin-3-yl)carbonyl]-l-glutamic Acid (**16h**)

^1^H NMR (400 MHz, DMSO-*d*_6_): δ 12.49 (br s, 1H), 10.01 (br s, 1H), 9.49 (br s, 1H), 8.80 (s, 1H), 8.71 (d, *J* = 4.0 Hz, 1H), 8.57 (d, *J* = 8.0 Hz, 1H), 8.14 (dd, *J* = 4.0 and 8.0 Hz, 1H overlapped with –NH), 7.67 (br d, *J* = 8.0 Hz,1H), 6.15 (s, 2H), 5.92 (s, 2H), 4.40–4.30 (m, 1H), 2.37 (t, *J* = 8.0 Hz, 2H), 2.10–2.00 (m, 1H), 2.00–1.87 (m, 1H); ^13^C NMR (100 MHz, DMSO-*d*_6_): δ 173.88, 173.40, 164.86, 161.30, 160.06, 155.34, 154.00, 153.31, 147.28, 137.24, 122.60, 110.43, 89.07, 51.89, 30.46, 26.03; MS (ES^+^) *m*/*z*: calcd for C_16_H_19_N_8_O_7_, 435.1, found, 435.1 (M + H)^+^; HRMS (ESI^–^): calcd for C_16_H_17_N_8_O_7_, 433.1220; found, 433.1219 (M – H)^−^; UPLC *t*_R_ = 5.94 min, 95.11%.

### *N*-(4-{[(2-Amino-4-hydroxy-6-oxo-1,6-dihydropyrimidin-5-yl)carbamoyl]amino}benzoyl)-l-glutamic Acid (**16i**)

^1^H NMR (400 MHz, DMSO-*d*_6_): δ 11.54 (br s, 2H), 8.85 (br s, 1H), 8.38 (d, *J* = 8.0 Hz, 1H), 7.78 (d, *J* = 8.0 Hz, 2H), 7.50 (d, *J* = 8.0 Hz, 2H), 6.78 (br s, 1H), 6.70 (br s, 2H), 4.40–4.34 (m, 1H), 2.35 (t, *J* = 8.0 Hz, 2H), 2.12–2.04 (m, 1H), 1.99–1.89 (m, 1H); ^13^C NMR (150 MHz, DMSO-*d*_6_): δ 174.05, 173.78, 166.29, 162.18, 154.82, 152.09, 143.57, 128.39, 126.27, 116.86, 91.30, 51.98, 30.53, 26.06; MS (ES^+^) *m*/*z*: calcd for C_17_H_19_N_6_O_8_, 435.1, found, 435.1 (M + H)^+^; HRMS (ESI^–^): calcd for C_17_H_17_N_6_O_8_, 433.1113; found, 433.1111 (M – H)^−^; UPLC *t*_R_ = 7.72 min, 96.7%.

### *N*-(4-{[(2-Amino-6-oxo-1,6-dihydropyrimidin-5-yl)carbamoyl]amino}benzoyl)-l-glutamic Acid (**16j**)

^1^H NMR (600 MHz, DMSO-*d*_6_): δ 9.63 (s, 1H), 8.46 (d, *J* = 7.2 Hz, 1H), 8.44 (s, 1H), 8.18 (br s, 3H), 7.84 (d, *J* = 9.0 Hz, 2H), 7.49 (d, *J* = 8.4 Hz, 2H), 4.40–4.36 (m, 1H), 2.35 (t, *J* = 7.2 Hz, 2H), 2.11–2.06 (m, 1H), 1.97–1.91 (m, 1H); ^13^C NMR (150 MHz, DMSO-*d*_6_): δ 173.96, 173.64, 166.08, 158.30, 152.05, 149.90, 142.26, 128.66, 127.27, 124.10, 118.16, 116.90, 51.93, 30.46, 25.98; MS (ES^+^) *m*/*z*: calcd for C_17_H_19_N_6_O_7_, 419.1, found, 419.1 (M + H)^+^; HRMS (ESI^–^): calcd, for C_17_H_17_N_6_O_7_, 417.1159; found, 417.1159 (M – H)^−^; UPLC *t*_R_ = 4.60 min, 97.9%.

### *N*-(4-{[(6-Amino-2,4-dioxo-1,2,3,4-tetrahydropyrimidin-5-yl)carbamoyl]amino}benzoyl)-l-glutamic Acid (**16k**)

^1^H NMR (600 MHz, DMSO-*d*_6_): δ 12.35 (br s, 2H), 10.37 (s, 1H), 10.17 (br s, 1H), 8.39 (d, *J* = 6.0 Hz, 1H), 7.79 (d, *J* = 12.0 Hz, 2H), 7.54 (br s, 2H), 6.75 (s, 1H), 6.18 (s, 2H), 4.38–4.34 (m, 1H), 2.34 (t, *J* = 6.0, Hz, 2H), 2.09–2.05 (m, 1H), 1.96–1.91 (m, 1H); ^13^C NMR (150 MHz, DMSO-*d*_6_): δ 173.97, 173.70, 166.16, 162.19, 154.74, 152.68, 149.92, 143.44, 128.33, 126.34, 116.70, 86.78, 51.91, 30.49, 26.01; MS (ES^–^) *m*/*z*: calcd for C_17_H_17_N_6_O_8_, 433.1, found, 433.1 (M – H)^−^; HRMS (ESI^–^): calcd for C_17_H_17_N_6_O_8_, 433.1113; found, 433.1108 (M – H)^−^; UPLC *t*_R_ = 6.80 min, 95.1%.

### *N*-(4-{[(4-Amino-6-oxo-1,6-dihydropyrimidin-5-yl)carbamoyl]amino}benzoyl)-l-glutamic Acid (**16l**)

^1^H NMR (300 MHz, DMSO-*d*_6_): δ 11.74 (s, 1H), 9.04 (s, 1H), 8.41 (d, *J* = 7.8 Hz, 1H), 7.80 (d, *J* = 8.7 Hz, 2H), 7.76 (s, 1H), 7.51 (d, *J* = 8.7 Hz, 2H), 7.11 (s, 1H), 6.37 (s, 2H), 4.41–4.34 (m, 1H), 2.35 (t, *J* = 7.4 Hz, 2H), 2.11–2.05 (m, 1H), 2.00–1.90 (m, 1H); ^13^C NMR (150 MHz, DMSO-*d*_6_): δ 173.96, 173.67, 166.20, 159.49, 153.82, 146.74, 143.31, 128.46, 126.42, 116.74, 98.45, 51.89, 30.46, 25.97; MS (ES^+^) *m*/*z*: calcd for C_17_H_19_N_6_O_7_, 419.1, found, 419.2 (M + H)^+^; HRMS (ESI^–^): calcd for C_17_H_17_N_6_O_7_, 417.1159; found, 417.1159 (M – H)^−^; UPLC *t*_R_ = 4.96 min, 98.3%.

### *N*-[(4-{[(2,4-Diamino-6-oxo-1,6-dihydropyrimidin-5-yl)carbamoyl]amino}phenyl)sulfonyl]-l-glutamic Acid (**19**)

To a mixture of amine **17** (1.7 mmol) and DIEPA (2.0 mmol) in dry CH_2_Cl_2_ (20 mL) was added bis(trichloromethyl)carbonate (0.7 mmol) at 15 °C and the mixture was stirred for 30 min. The organic solvent was removed under reduced pressure to give the crude product isocyanate **18**. 4-Hydroxy-2,5,6-triaminopyrimidine sulfate (1.5 mmol) was suspended in water (2 mL) and mixed with a 1 N NaOH solution (5.0 mL). A solution of compound **18** (1.7 mmol) in 5 mL of acetonitrile was added dropwise. After stirring at room temperature for 12 h, the solvent were removed under reduced pressure. The mixture was adjusted to pH = 2 with 2 N HCl and stirred for 10 min. The solid was filtered off and washed with water. After drying, the crude product was purified by Al_2_O_3_ (eluted with CH_2_Cl_2_/CH_3_OH/HCOOH = 50/48/2) yielded the *tert*-butyl ester of **19** as a pale yellow solid. A mixture of *tert*-butyl ester (0.15 mmol) in 5 mL of TFA was stirred at room temperature for 10 min. TFA were removed under reduced pressure and the residue was washed with acetonitrile to give the desired diacid **19** as a pale brown solid. ^1^H NMR (300 MHz, DMSO-*d*_6_): δ 8.99 (br s, 1H), 7.89 (d, *J* = 9.0 Hz,, 1H), 7.63–7.56 (m, 4H), 6.85 (s, 1H), 6.71 (br s, 2H), 6.26 (br s, 2H), 3.70 (td, *J* = 12.0, 6.0 Hz, 1H), 2.21 (t, *J* = 6.0 Hz, 2H), 1.90–1.76 (m, 1H), 1.75–1.50 (m, 1H); ^13^C NMR (150 MHz, DMSO-*d*_6_): δ 173.54, 172.59, 160.22, 152.84, 144.22, 132.58, 127.68, 117.03, 89.08, 54.71, 29.58, 27.44; MS (ES^+^) *m*/*z*: calcd for C_16_H_20_N_7_O_8_S, 470.1, found, 470.2 (M + H)^+^; HRMS (ESI^–^): calcd for C_16_H_18_N_7_O_8_S, 468.0938; found, 468.0943 (M – H)^−^; UPLC *t*_R_ = 5.23 min, 96.6%.

### *N*-(4-{2-[(2,4-Diamino-6-oxo-1,6-dihydropyrimidin-5-yl)amino]-2-oxoethyl}benzoyl)-l-glutamic Acid (23)

To a solution of acid **20** (2.0 mmol) in dry CH_2_Cl_2_ was added thionyl chloride (4.0 mmol) and pyridine (one drop). The resultant solution was then refluxed at 55 °C for 1.5 h. Solvent were removed under reduced pressure and the crude product **21** was used directly for the next step without further purification. The synthetic route for the preparation of the final product diacid **23** starting from **21** is like that of **16**.

^1^H NMR (400 MHz, DMSO-*d*_6_): δ 8.54 (s, 1H), 8.49 (d, *J* = 8.0 Hz, 1H), 7.81 (d, *J* = 8.0 Hz, 2H), 7.43 (d, *J* = 8.0 Hz, 2H), 6.20 (s, 2H), 5.61 (s, 2H), 4.42–4.36 (m, 1H), 3.62 (s, 2H), 2.35 (t, *J* = 8.0 Hz, 2H), 2.12–2.04 (m, 1H), 2.00–1.91 (m, 1H); ^13^C NMR (150 MHz, DMSO-*d*_6_): δ 174.04, 173.57, 169.62, 166.42, 160.61, 159.79, 153.40, 140.40, 131.96, 129.13, 127.27, 89.82, 52.08, 42.01, 30.61, 26.12; MS (ES^+^) *m*/*z*: calcd for C_18_H_21_N_6_O_7_, 433.2, found, 433.2 (M + H)^+^; HRMS (ESI^–^): calcd for C_18_H_19_N_6_O_7_, 431.1315; found, 431.1315 (M – H)^−^; UPLC *t*_R_ = 5.21 min, 99.7%.

### Protein Expression and Purification for Structural Biology Studies

The vector pET14b (Novagen, Madison, USA) was utilized for the expression of MTHFD2. The cDNA fragment encoding MTHFD2 residues 36–350 was cloned with an *N*-terminal 6×-histidine tag followed by a thrombin cleavage site. Expression was induced in the bacterial strain BL21(DE3) and continued for 6 h. Harvested cell pellets stored at −80 °C was subsequently lysed by sonication in lysis buffer (50 mM Tris, pH 7.8, 250 mM NaCl). MTHFD2 proteins were purified using a HisTrap HP column (Cytiva, Marlborough, USA). The eluate containing MTHFD2 protein was then exchanged into thrombin cleavage buffer (20 mM Tris, pH 8.2, 150 mM NaCl, 2.5 mM CaCl_2_) and subjected to overnight digestion with thrombin protease at 4 °C. The MTHFD2 protein solution was finally concentrated to 7 mg/mL with the addition of 1 mM TCEP.

The vector pET28a (Novagen, Madison, USA) was employed for the expression of MTHFD1. The cDNA fragment encoding human MTHFD1 residues 1–301 was cloned with a C-terminal 6x-histidine tag. Expression was induced in the bacterial strain BL21(DE3) and continued for 6 h. MTHFD1 proteins were purified with a HisTrap HP column. After washing, eluant containing MTHFD1 proteins was exchanged to the final buffer (20 mM Tris, pH 7.3, 250 mM NaCl) using a HiPrep 26/10 desalting column. The MTHFD1 protein solution was finally concentrated to 7 mg/mL with the addition of 1 mM TCEP and NADP^+^.

### Crystallization

Crystals of MTHFD2 or MTHFD1 in complex with LY374571, **16a**, **16d**, **16e** and **16g** were prepared using the cocrystallization method. MTHFD2 or MTHFD1 proteins were incubated with 1 to 3 mM of various compounds on ice for 30 min. Crystals of MTHFD2 were obtained by the hanging-drop vapor-diffusion method, where 1 μL of protein was mixed with 1 μL of a reservoir solution containing 23% isopropanol, 0.1 M bis-Tris, pH 7.1, 0–8% PEG200, 5–20% PEG400, 0–12% PEG1000. For MTHFD1 crystals, the protein/compound mixture were added to a reservoir solution consisting of 0.09 M Morpheus NPS Mix, 0.01 M Morpheus Buffer System 2, pH 7.2 or 7.5 and 31–42% Morpheus Precipitant Mix 4 (Molecular Dimensions). After 1 week, the crystals were grown at 18 °C. Crystals of MTHFD2 were flash-frozen in liquid nitrogen with the addition of DMSO to the drop as a cryoprotectant, while MTHFD1 crystals were flash-frozen directly without cryoprotectant.

### Structure Determination

Diffraction data were obtained at the NSRRC, Taiwan (beamline TLS15A1 and TPS05A) and SPring-8 (beamline BL44XU), Japan. The data were processed using HKL2000^55^ software. The molecular replacement method MOLREP^56^ of the CCP4^57^ program suites was applied to determine the protein structures, using the MTHFD2 structure (PDB 5TC4) and MTHFD1 structure (PDB 1DIG) as the template models. The programs PHENIX^58^ and COOT^59^ were used for subsequent refinement and model building. Figures of structures were generated by PyMOL (Schrödinger, LLC.).

### Cell Culture

MOLM-13, MOLM-14, HSC-3, and HuH-7 human cancer cells were purchased from Japanese Collection of Research Bioresources Cell Bank (Osaka, Japan). RS4; 11, FaDu, NCI-H2030, and MIA PaCa-2 were purchased from American Type Culture Collection (Virginia, US). HL-60, CCRF-CEM, K-562, SW620, LoVo, Hep G2, A549, and AsPC-1 human cancer cell lines were purchased from Bioresource Collection and Research Center (BCRC) (Hsinchu, Taiwan). All cell lines were cultured according to the manufacturer’s protocol and maintained in a humidified incubator with 5% CO_2_ at 37 °C.

### Protein Expression and Purification for Activity Assays

The preparation of purified human MTHFD2 and MTHFD1 proteins followed the protocol described by Gustafsson et al.^29^ Briefly, a 36–350 amino acid fragment of human MTHFD2 and a 1–306 amino acid fragment of human MTHFD1 were constructed using the pBacPAK8-MTGFP-His vector and overexpressed in insect cells. The C-terminal-His-tagged MTHFD2 and MTHFD1 proteins were then purified using a HisTrap-HP column (Cytiva, Marlborough).

### MTHFD1 and MTHFD2 Activity Assays

These assays were performed and modified by Mejia and MacKenzie^60^ Purified MTHFD2 and MTHFD1 were preincubated with 0.4 mM NAD^+^ and 2 mM NADP^+^, respectively. After a 10 min incubation, the MTHFD2 inhibitor was added and incubated for an additional 10 min. MTHFD1 and MTHFD2 enzymatic reactions were carried out for 10 min after the respective assay buffer was added. The MTHFD1 assay buffer included 30 mM potassium phosphate, pH 7.3, 0.3 mM tetrahydrofolate, 2.5 mM formaldehyde, 6 mM MgCl_2_, and MTHFD2 assay buffer included 30 mM potassium phosphate, pH 7.3, 0.15 mM tetrahydrofolate, 2.5 mM formaldehyde, 6 mM MgCl_2_. Finally, the enzymatic reaction was terminated by adding HCl (final 0.18 N) and the absorbance was measured at 350 nm.

### Cell Growth Inhibition Assays

Suspension and adherent cells were seeded in 96-well- and 24-well plates, respectively. After culturing overnight, cells were treated with MTHFD2 inhibitors, such as LY374571, **16e**, and **16a**, for 72 h. Suspension cells were analyzed using the MTT assay, while adherent cells were examined using methylene blue staining assay. The efficacy of the compounds was evaluated through GI_50_ analysis, which determines the concentration of a compound that inhibits 50% of cell growth.

### Drugs Combination Assays

Seeded MOLM-14 cells were treated with increased concentrations of Alimta combined with either **16a** or **16e**. After 72 h of incubation, cell viability was assessed using the MTT assay. The combination index was analyzed by Calcusyn software (Version 1.1.1). A combination index score of less than 1 indicated a synergistic effect, a score of 1 indicated an additive effect, and a score greater than 1 indicated an antagonistic effect.

### Pharmacokinetic Studies

Male ICR mice aged 6–8 weeks (Biolasco, Taiwan) were intravenously administered a single dose of either 2 mg/kg LY374571 or 2 mg/kg **16e**. After drug treatment, mice were sacrificed at 0.003, 0.083, 0.25, 0.5, 1, 2, 4, 6, 8, and 24 h, with three mice per group. Plasma samples were collected by cardiac puncture and analyzed using LC–MS/MS to obtain the data of moderate plasma half-life (*T*_1/2_), area under the curve–time curve from 0 to infinity (AUC _(**0-inf**)_), total body clearance rate (CL), and volume of distribution (*V*_ss_).

### In Vivo Anticancer Studies

All mice (NOD/SCID, female, 9–10 weeks) were purchased from BioLASCO (Taiwan) and experiments were performed in strict accordance with the recommendations in the guidelines for the Care and Use of Laboratory Animals of the National Health Research Institutes (Miaoli, Taiwan). The Institutional Animal Care and Use Committee of the National Health Research Institutes approved the animal protocol (protocol no. 107085). MOLM-14 cells were subcutaneously implanted in the flank region of mice. Drug treatments were started when the tumor size reached ∼100 mm^3^ (D1). Tumor size and weight change were monitored twice weekly until the study finished. Tumor size (mm^3^) was calculated from (*w*^2^ × *l*)/2. Where *w* = width and *l* = length in mm of the tumor. Tumor growth inhibition (TGI) was used to evaluate the antitumor efficacy and was calculated as follows: TGI = {1 – [(mean volume of treatment group at day 7 or day 14 – mean volume of treatment group at day 1)/(mean volume of control group at day 7 or day 14 – mean volume of control group at day 1)] × 100%}.

### Statistical Analysis

Data was expressed as the mean ± SD. Student’s *t*-test was used to examine differences between the control and test groups. Differences were considered significant at *p* < 0.05. All statistics were performed using SigmaStat (Jandel Scientific, Palo Alto, CA) Ancillary Information.

## Supporting Information Available

The 2*F*_0_ – *F*_c_ electron density maps of ligands; The docking study of MTHFD2 with **19**; Comparison of the growth inhibitory effect of compound **16e** on MOLM-14 and normal cells; ^1^H and ^13^C NMR spectrum of **16a–16L**, **19**, **23**; (PDF)
Molecular formula strings (CSV)

## Notes

PDB ID Codes 9IS9 (MTHFD2/LY374571); 9ISC (MTHFD2/**16a**); 9ITA (MTHFD2/**16d**); 9IT3 (MTHFD2/**16e**); 9IT6 (MTHFD2/**16g**); 9ISE (MTHFD1/LY374571); 9ITD (MTHFD1/**16a**); 9IUO (MTHFD1/**16d**); 9ISL (MTHFD1/**16e**); 9ISR (MTHFD1/**16g**). Authors will release the atomic coordinates and experimental data upon article publication.

## Abbreviations

10-CHO-THF: 10-formyl tetrahydrofolate
FLT3-ITD: FLT3 internal tandem duplication
MTHFD2: methylenetetrahydrofolate dehydrogenase/cyclohydrolase 2
SAR: structure–activity relationship
SCID: severe combined immunodeficiency
