# Supplementary material for: Development of Potent and Selective Inhibitors of Methylenetetrahydrofolate Dehydrogenase 2 for Targeting Acute Myeloid Leukemia: SAR, Structural Insights, and Biological Characterization
Source: J Med Chem. 2024 Nov 26;67(23):21106–25. doi: 10.1021/acs.jmedchem.4c01775 (PMC11647893; doi:10.1021/acs.jmedchem.4c01775)
Supplement: Supplementary file 1 — jm4c01775_si_001.pdf [file jm4c01775_si_001.pdf]

Supporting Information

Development of Potent and Selective Inhibitors of Methylenetetrahydrofolate Dehydrogenase 2 (MTHFD2) for Targeting Acute Myeloid Leukemia: SAR, Structural Insights, and Biological Characterization

Hsin-Huei Chang, [†#] *Lung-Chun Lee,*[†#] *Tsu Hsu,* [†] *Yi-Hui Peng,* [†] *Chih-Hsiang Huang, Teng-Kuang Yeh,* [†] *Cheng-Tai Lu,*[†] *Zih-Ting Huang,*[†] *Ching-Cheng Hsueh,*[†] *Fang-Chun Kung,*[†] *Li-Mei Lin,*[†] *Yu-Chen Huang,*[†] *Yi-Hsin Wang,*[†] *Li-Hsuan Li,* [†§] *Ya-Chu Tang*[†] *Ling Chang,*[†] *Chih-Chien Hsieh,*[†] *Weir-Torn Jiaang,* [†*] *Ching-Chuan Kuo,* [†*] *and Su-Ying Wu*[†*]

[†] Institute of Biotechnology and Pharmaceutical Research, National Health Research Institutes, 35, Keyan Road, Zhunan Town, Miaoli County 350, Taiwan, R.O.C.

[§]Institute of Biotechnology, National Tsing Hua University, Hsinchu, Taiwan, R.O.C.

[#] Hsin-Hui Chang and Lung-Chun Lee contributed equally

Corresponding author:

Su-Ying Wu, Email:suying@nhri.org.tw

Ching-Chuan Kuo, Email:cckuo@nhri.org.tw

Weir-Torn Jiaang, Email:wtjiaang@nhri.org.tw

Table of Contents

Figure S-1. The 2F_o-F_c electron density maps of ligands ……………………………S3
Figure S-2. The docking study of MTHFD2 with 19 ....S4
Figure S-3. Comparison of the growth inhibitory effect of compound 16e on MOLM-14 and normal cells……………………..S5
Figure S-4. ^{1}H and ^{13}C NMR spectrum of 16a...S6
Figure S-5. ^{1}H and ^{13}C NMR spectrum of 16b ……………………………………...S7
Figure S-6. ^{1}H and ^{13}C NMR spectrum of 16c ………………………………….......S8
Figure S-7. ^{1}H and ^{13}C NMR spectrum of 16d ………………………………….......S9
Figure S-8. ^{1}H and ^{13}C NMR spectrum and HPLC trace of 16e………………...…S11
Figure S-9. ^{1}H and ^{13}C NMR spectrum of 16f ……………………………………S12
Figure S-10. ^{1}H and ^{13}C NMR spectrum of 16g…………………………….........S13
Figure S-11. ^{1}H and ^{13}C NMR spectrum of 16h……………………………………S14
Figure S-12. ^{1}H and ^{13}C NMR spectrum of 16i ………………………………….....S15
Figure S-13. ^{1}H and ^{13}C NMR spectrum of 16j……………………………………...S16
Figure S-14. ^{1}H and ^{13}C NMR spectrum of 16k……………………………………S17
Figure S-15. ^{1}H and ^{13}C NMR spectrum of 16l……………………………………...S18
Figure S-16. ^{1}H and ^{13}C NMR spectrum of 19 …………………………………....S19
Figure S-17. ^{1}H and ^{13}C NMR spectrum of 23…………………………………......S20

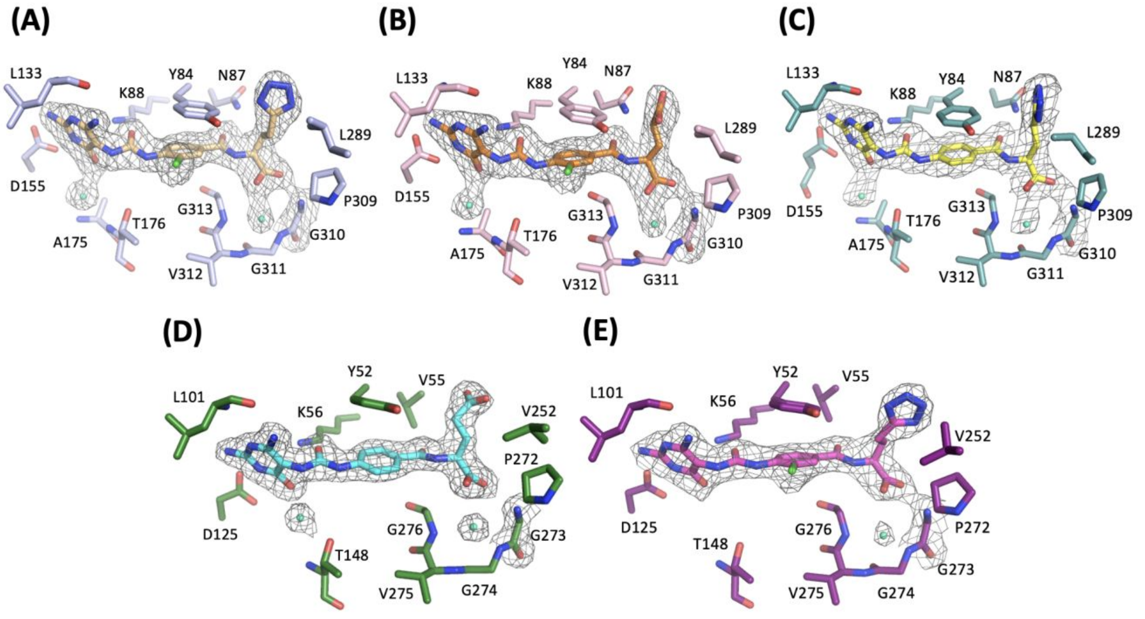

Figure S-1. The $2F_o$-F_c electron density maps of ligands and surrounded water molecules. (A) The $2F_o$-F_c electron density map contoured around **16g** (1.0 σ) calculated for the MTHFD2/**16g**/NAD$^+$ structure (PDB 9IT6, resolution=2.04 Å). (B) The $2F_o$-F_c electron density map contoured around **16e** (1.0 σ) calculated for the MTHFD2/**16e**/NAD$^+$ structure (PDB 9IT3, resolution=2.06 Å). (C) The $2F_o$-F_c electron density map contoured around **16a** (0.9 σ) calculated for the MTHFD2/**16a**/NAD$^+$ structure (PDB 9ISC, resolution= 2.54 Å). (D) The $2F_o$-F_c electron density map contoured around LY374571 (1.0 σ) calculated for the MTHFD1/LY374571/NADP$^+$ structure (PDB 9ISE, resolution= 1.99 Å). (E) The $2F_o$-F_c electron density map contoured around **16g** (0.9 σ) calculated for the MTHFD1/**16g**/NADP$^+$ structure (PDB 9ISR, resolution=2.50 Å). NAD$^+$ or NADP$^+$ is omitted for clarity.

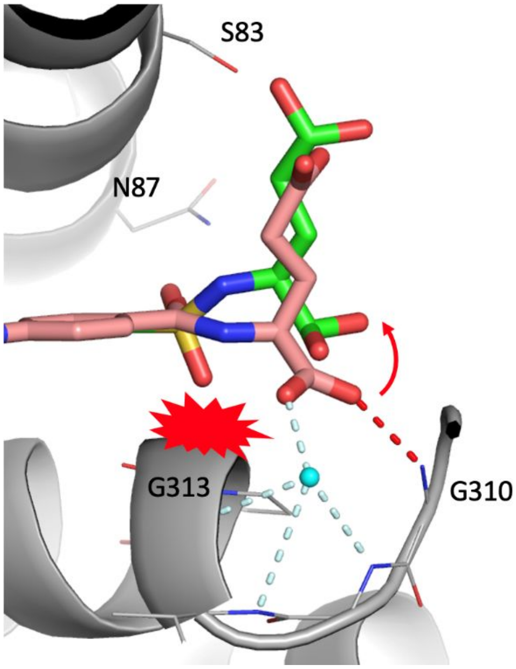

Figure S-2. The docking study depicting substitution of the amide linker of LY374571 in MTHFD2/LY374571 (PDB 9IS9, gray/pink) with a sulfonyl group (compound **19**, green) within the binding site of MTHFD2. Sulfonyl moiety of compound **19** introduces steric clashes with Gly313 and induces a shift in the α-carboxylic acid group, leading to the disruption of its hydrogen bond with the main chain of G310 and a diminished water-mediated hydrogen bond network involving Gly311, Val312, and Gly313.

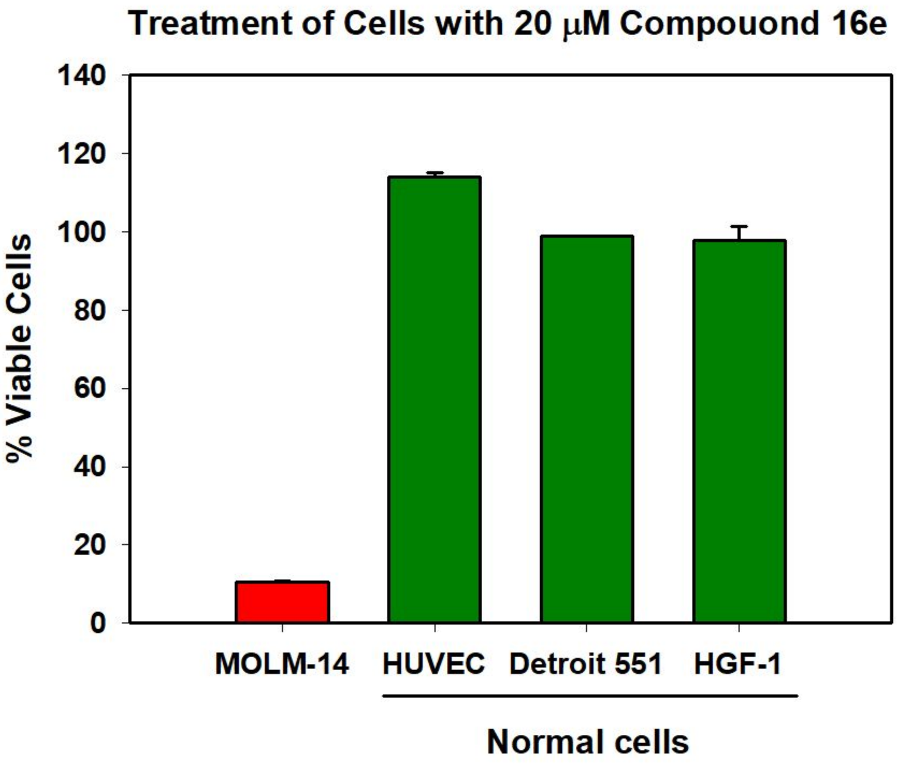

Figure S-3. Comparison of the growth inhibitory effect of compound **16e** on MOLM-14 and normal cells. MOLM-14 cells and three normal cell lines, including HUVEC (human umbilical vein endothelial cells), Detroit 551 (normal human skin fibroblasts), and HGF-1 (normal human gingival fibroblasts), were treated with 20 μM compound **16e** for 72 hours. Cell viability was assessed using the MTT assay.

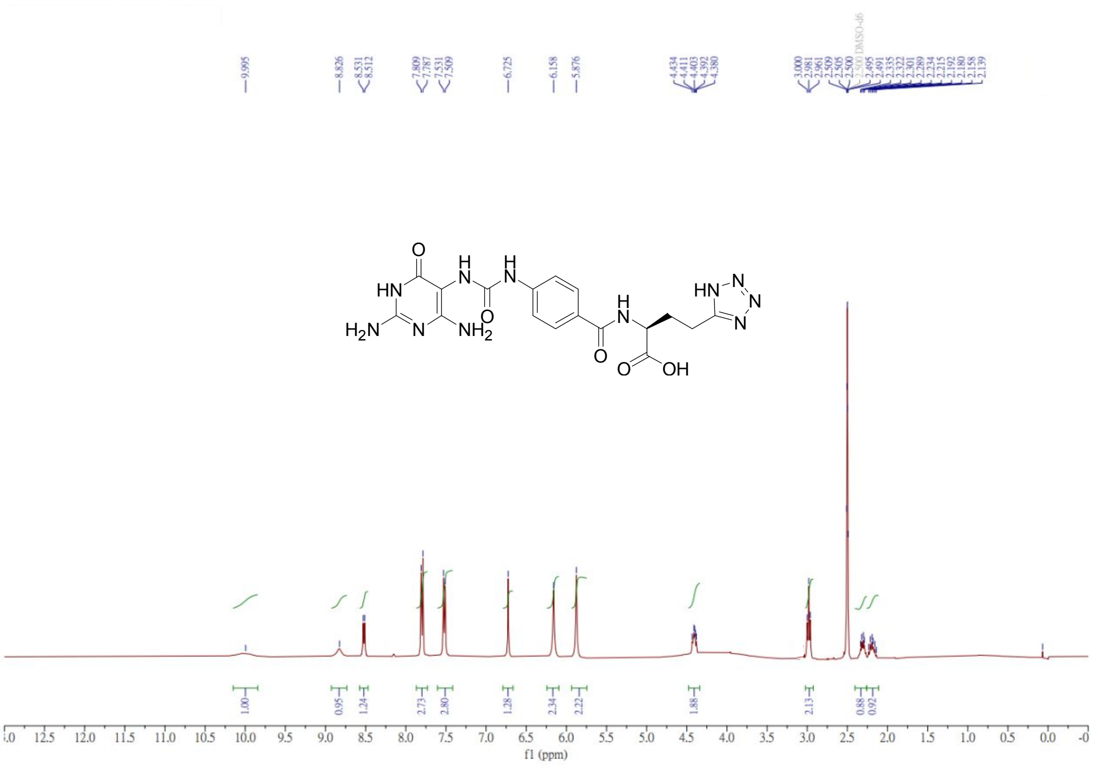

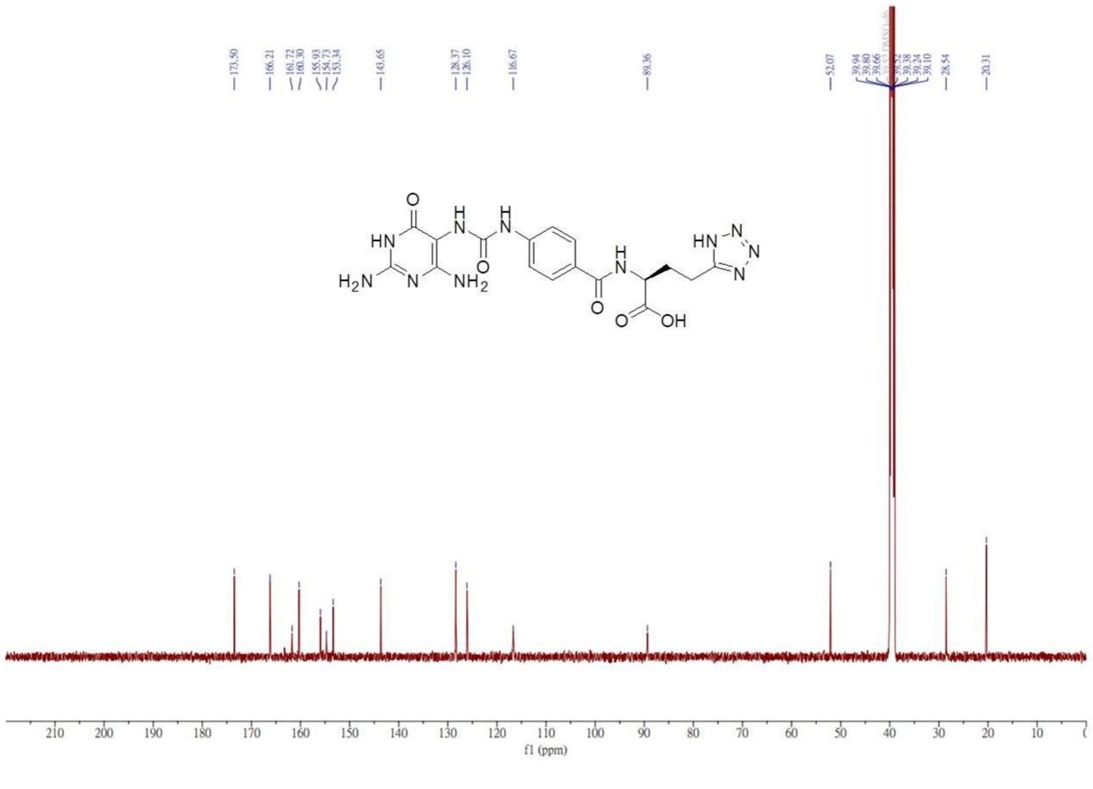

Figure S-4. ^{1}H and ^{13}C NMR spectrum of **16a** in DMSO-d_6.

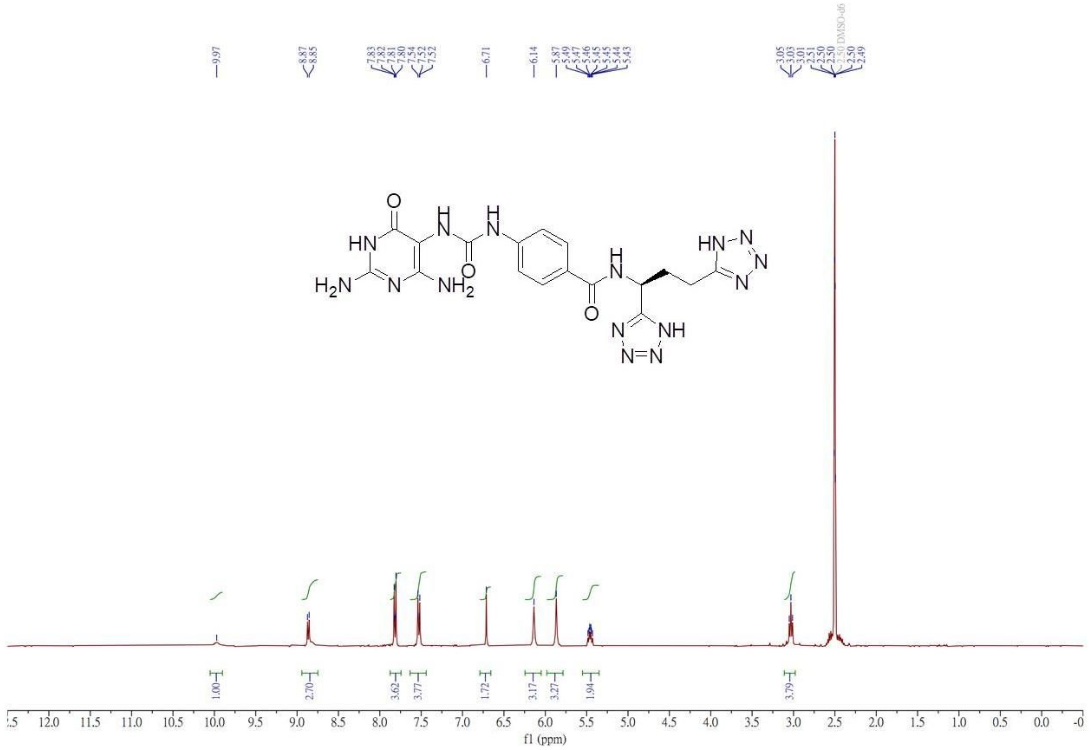

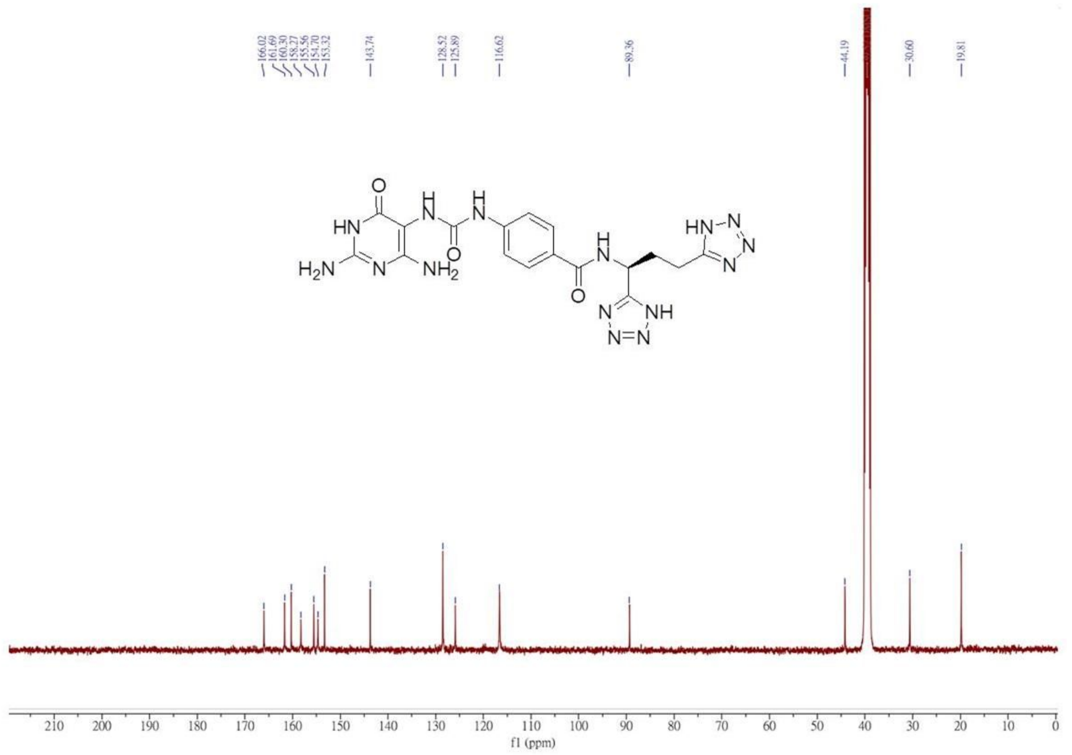

Figure S-5. ^{1}H and ^{13}C NMR spectrum of **16b** in DMSO-d_6.

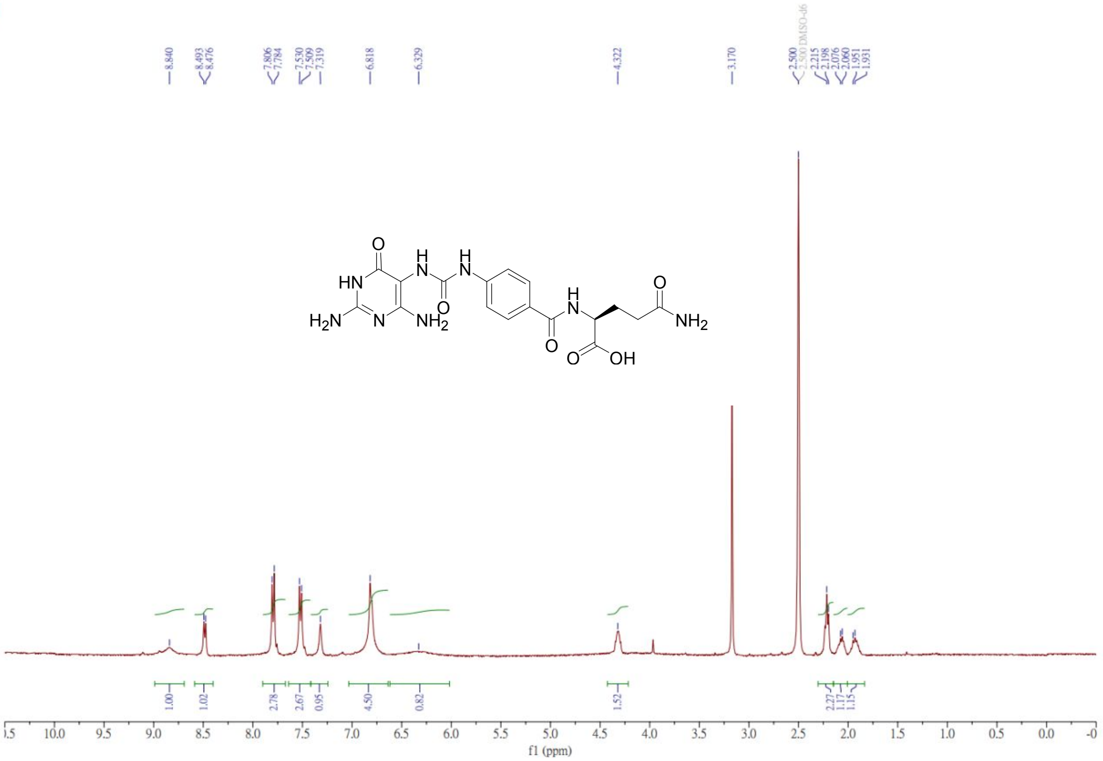

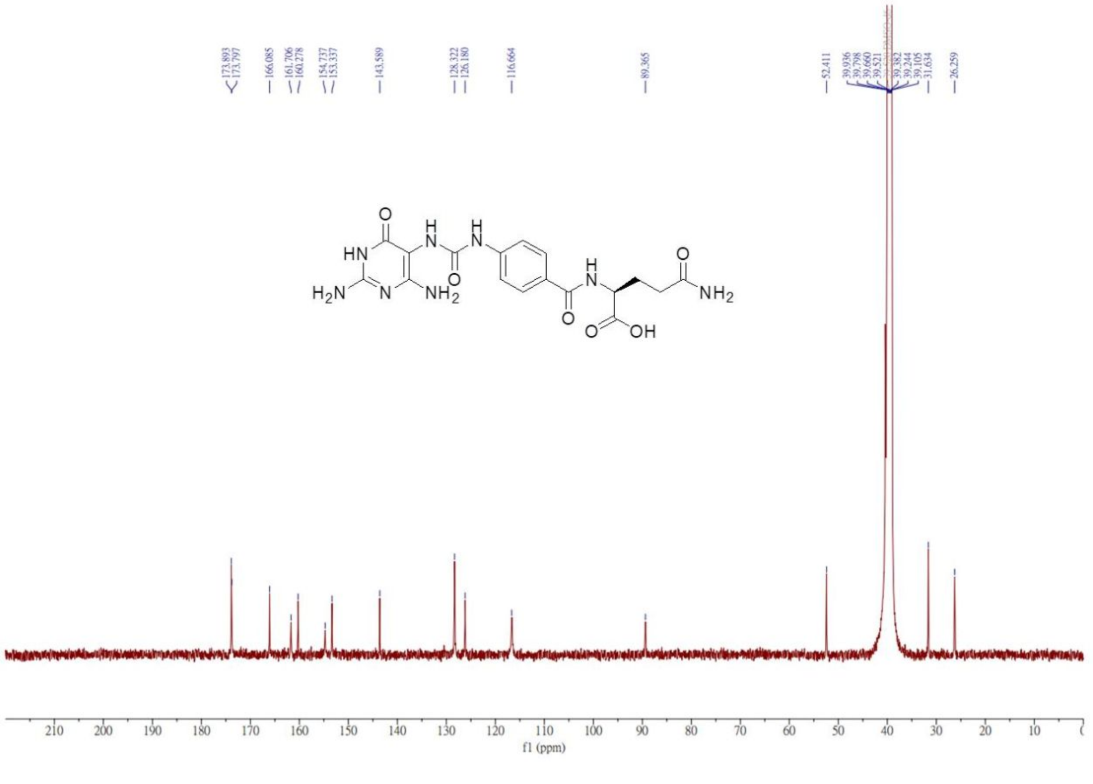

Figure S-6. ^{1}H and ^{13}C NMR spectrum of **16c** in DMSO-d_6.

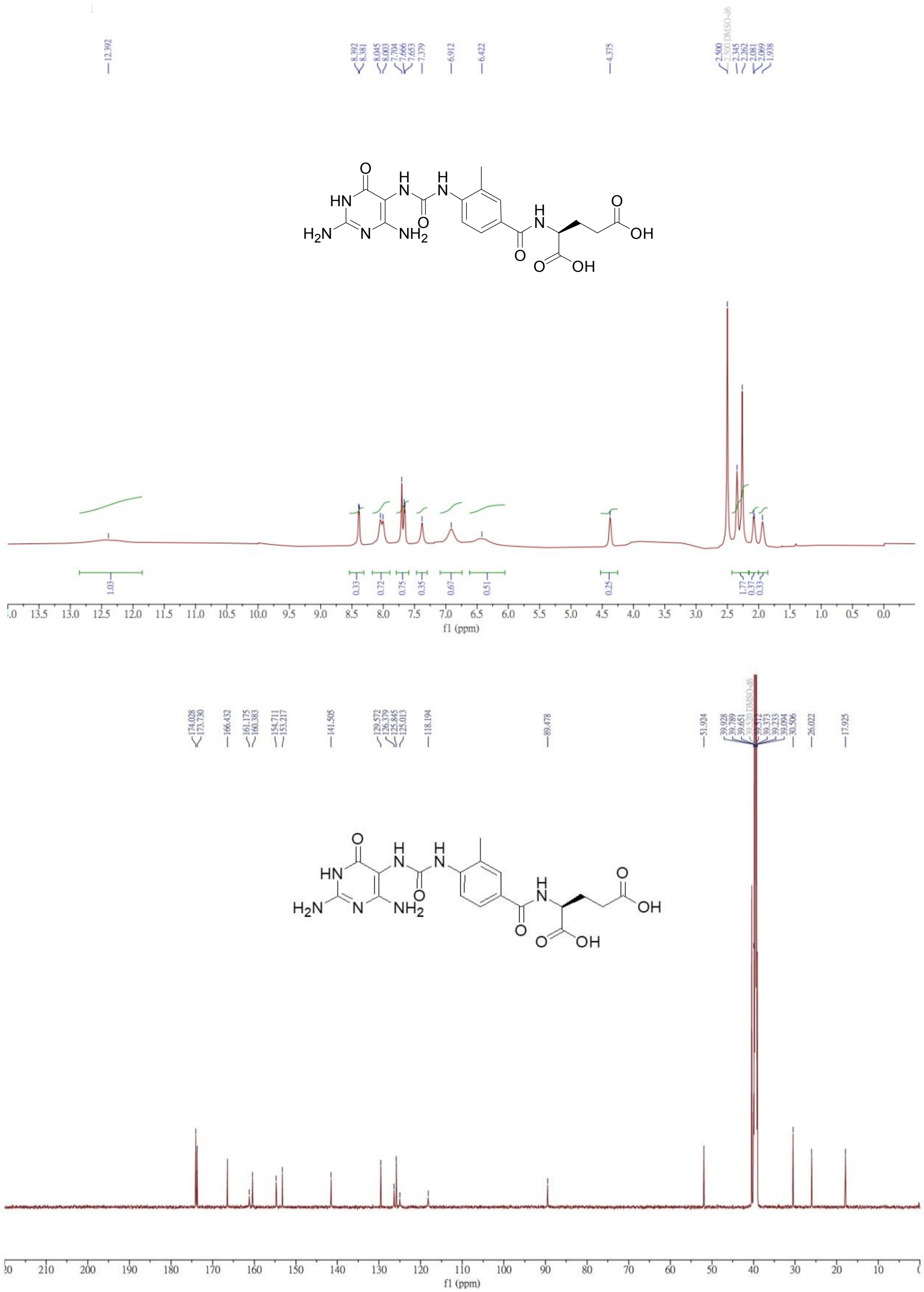

Figure S-7. ^{1}H and ^{13}C NMR spectrum of **16d** in DMSO-d_6.

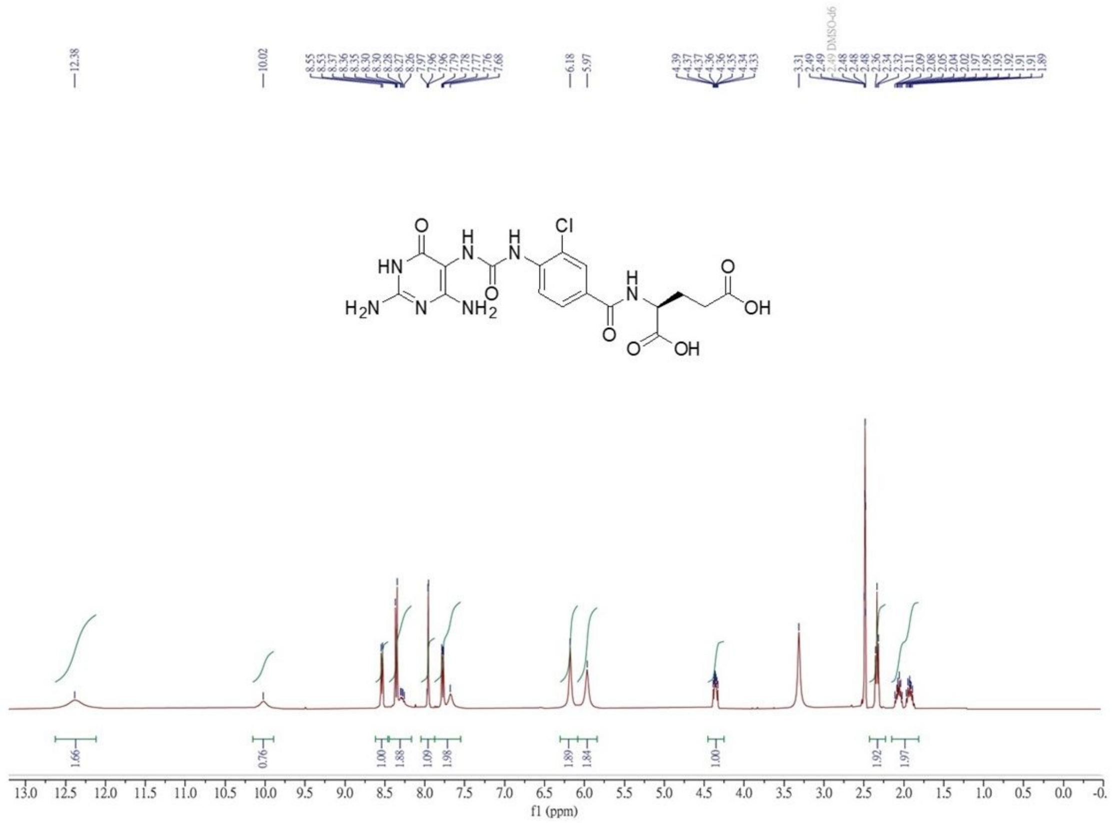

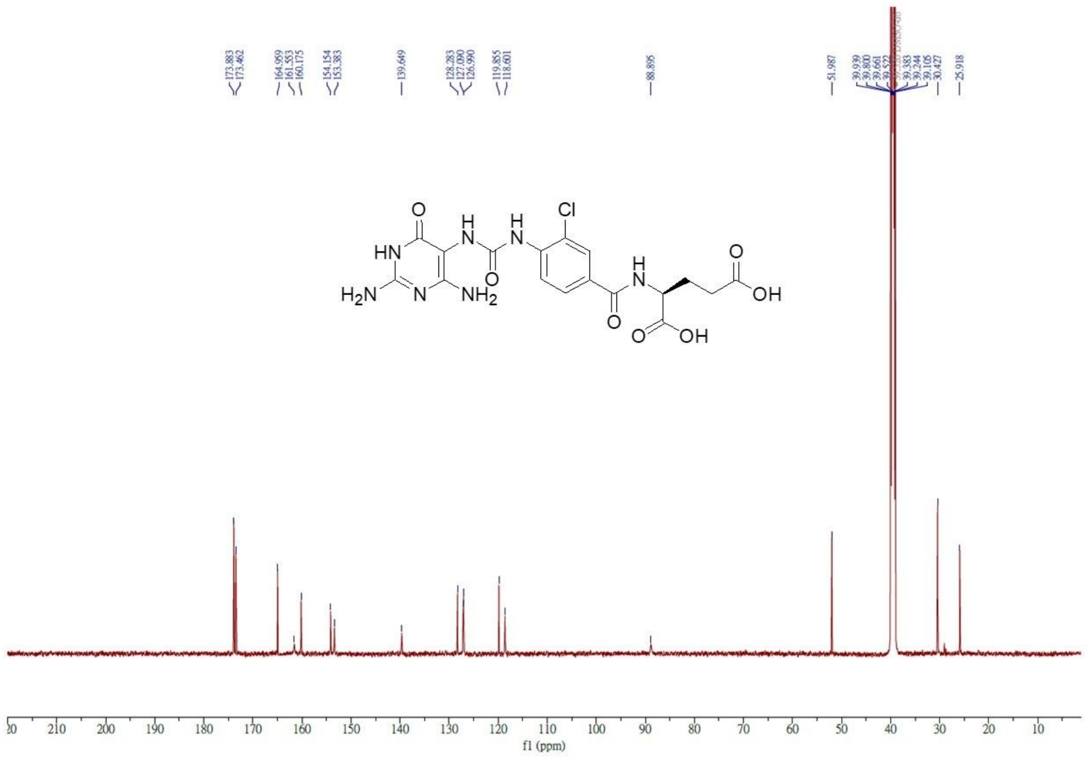

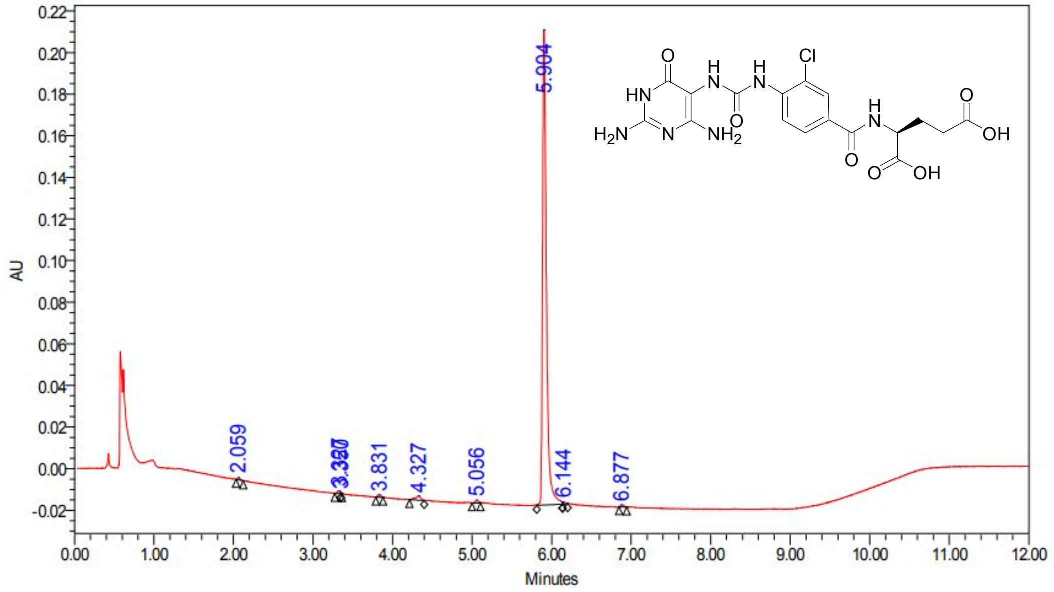

	RT	Height	Area	% Area
1	2.059	1161	2900	0.36
2	3.327	1323	3466	0.44
3	3.350	656	359	0.05
4	3.831	1177	3265	0.41
5	4.327	2031	10429	1.31
6	5.056	1343	3386	0.43

	RT	Height	Area	% Area
7	5.904	228566	766645	96.30
8	6.144	1081	2589	0.33
9	6.877	1055	3044	0.38

Figure S-8. ^{1}H and ^{13}C NMR spectrum and HPLC trace of **16e** in DMSO-d_6.

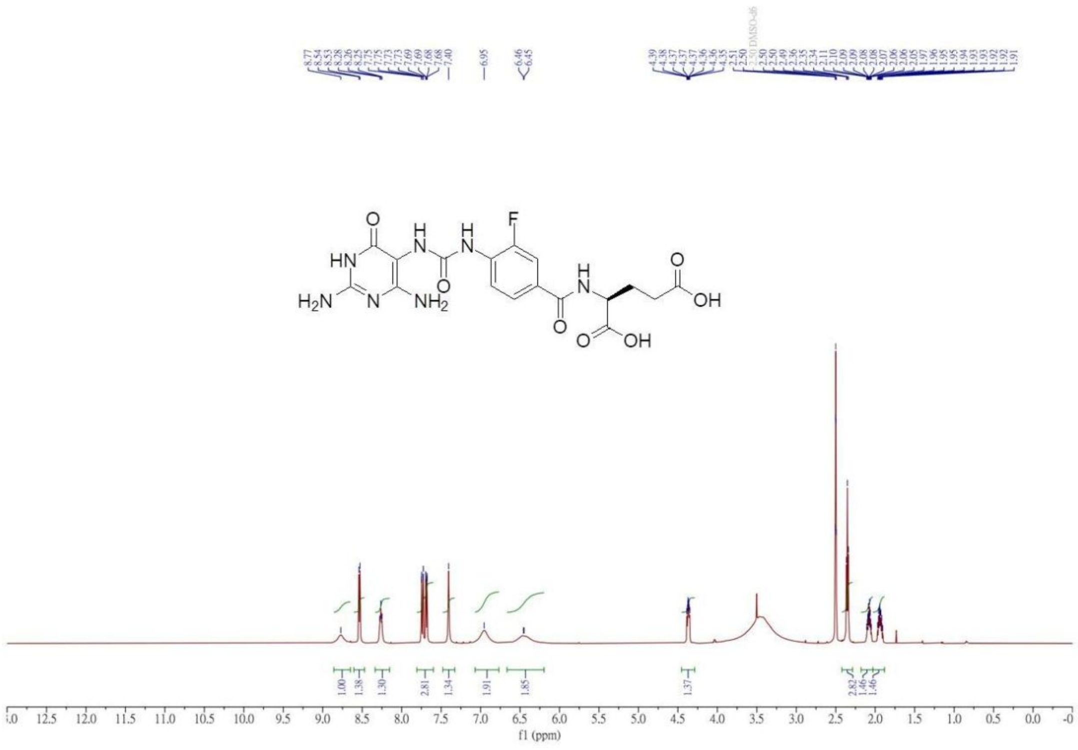

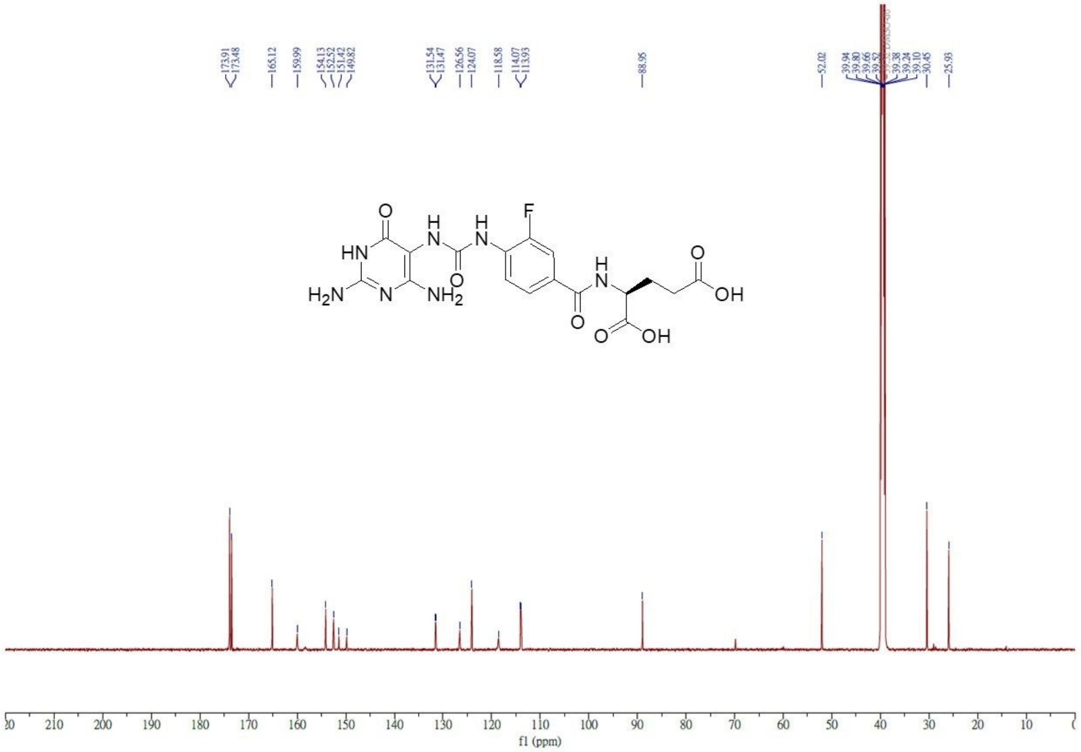

Figure S-9. ^{1}H and ^{13}C NMR spectrum of **16f** in DMSO-d_6.

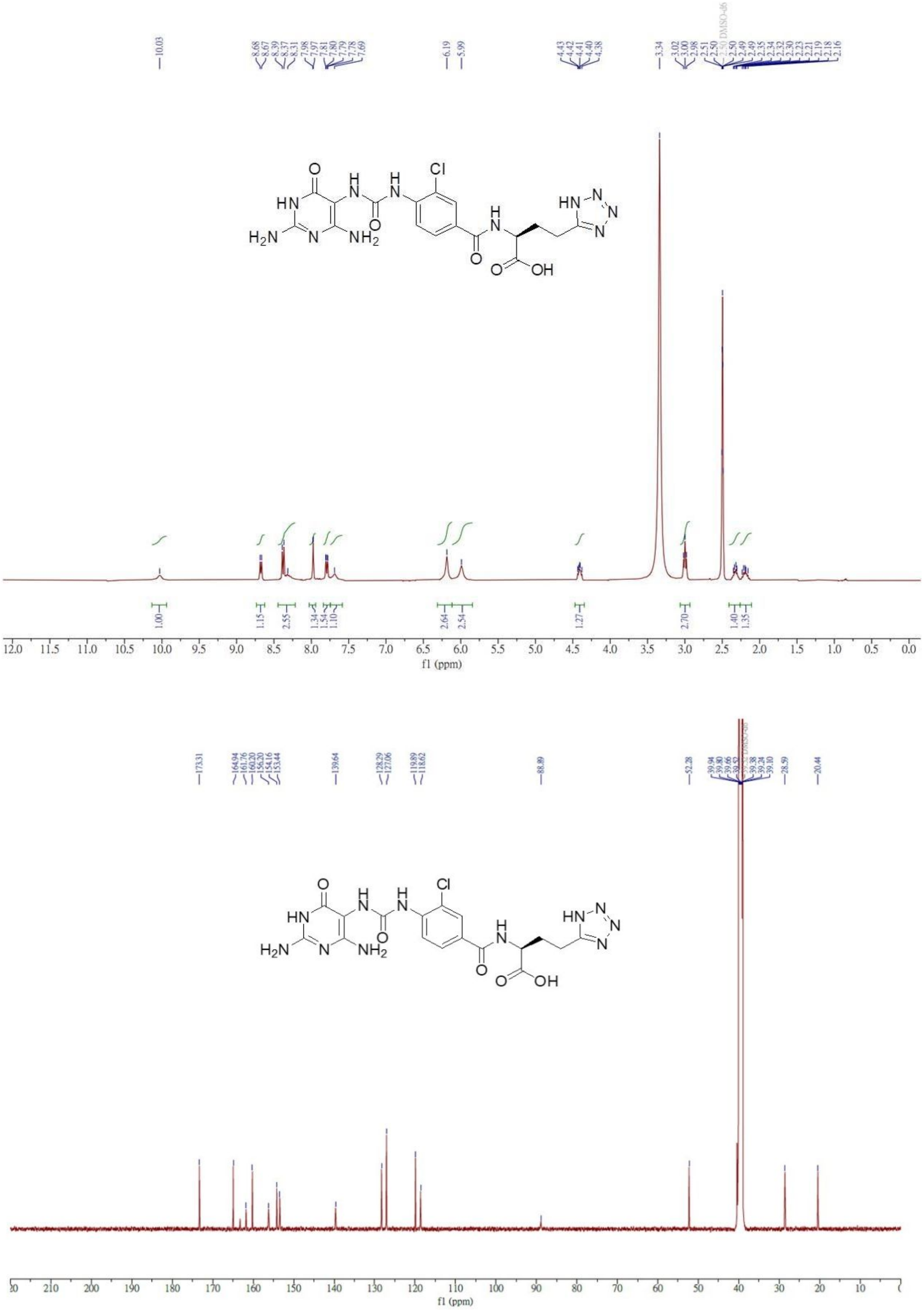

Figure S-10. ^{1}H and ^{13}C NMR spectrum of **16g** in DMSO-d_6.

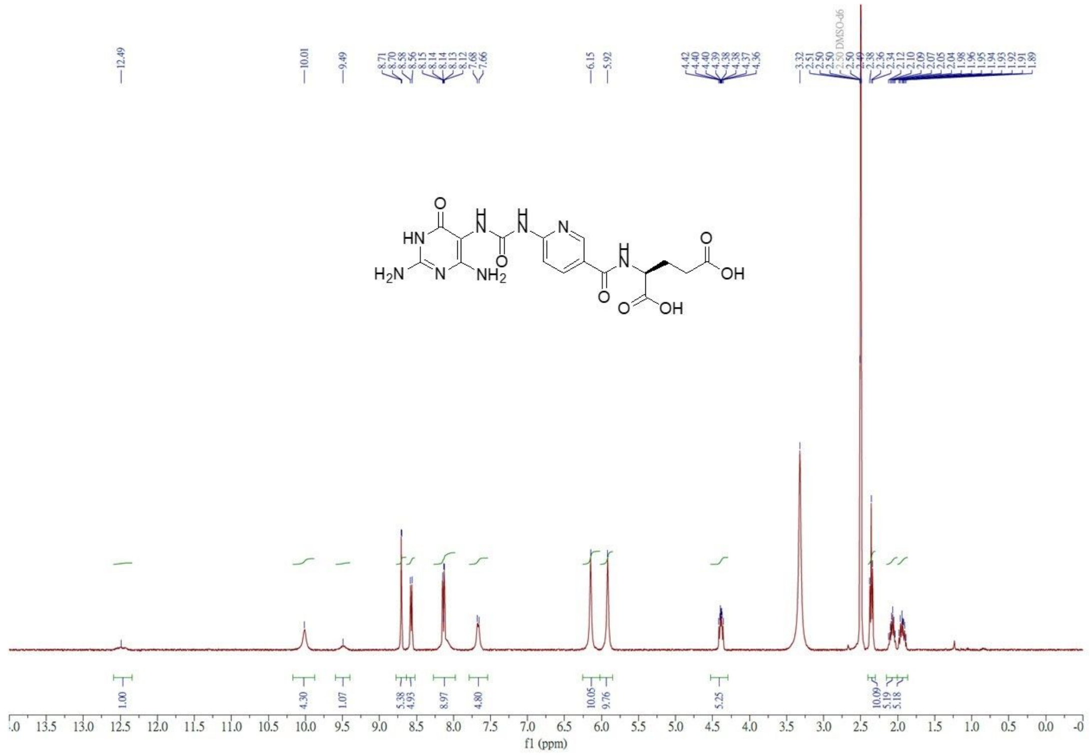

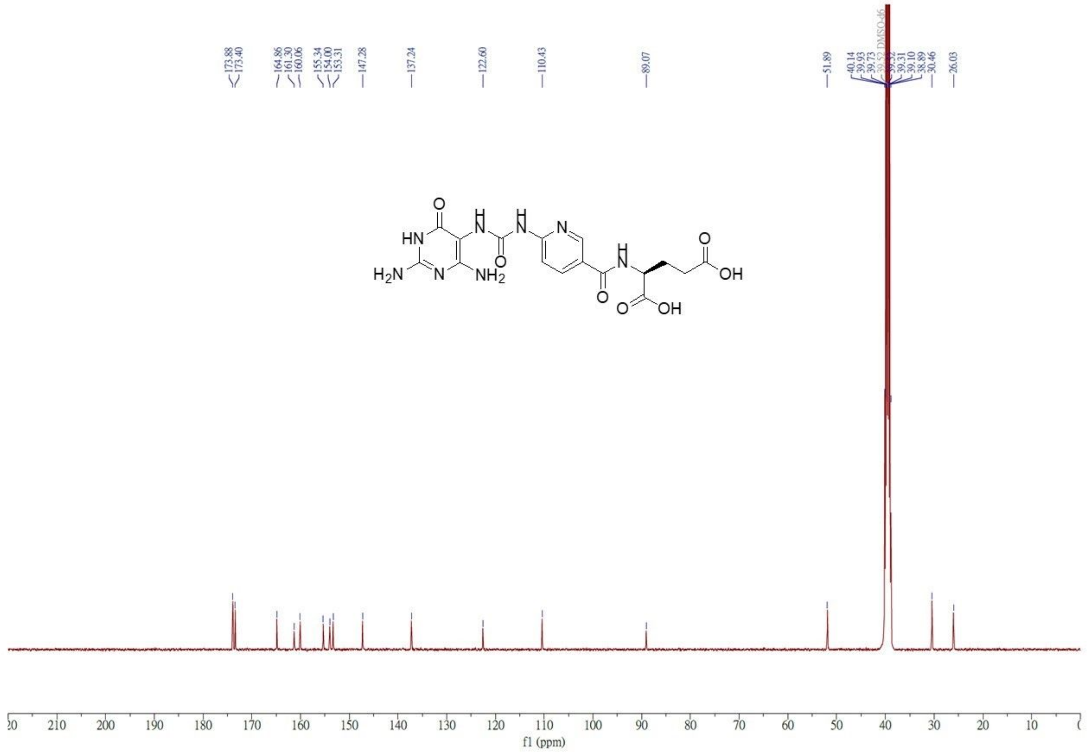

Figure S-11. ^{1}H and ^{13}C NMR spectrum of **16h** in DMSO-d$_6$.

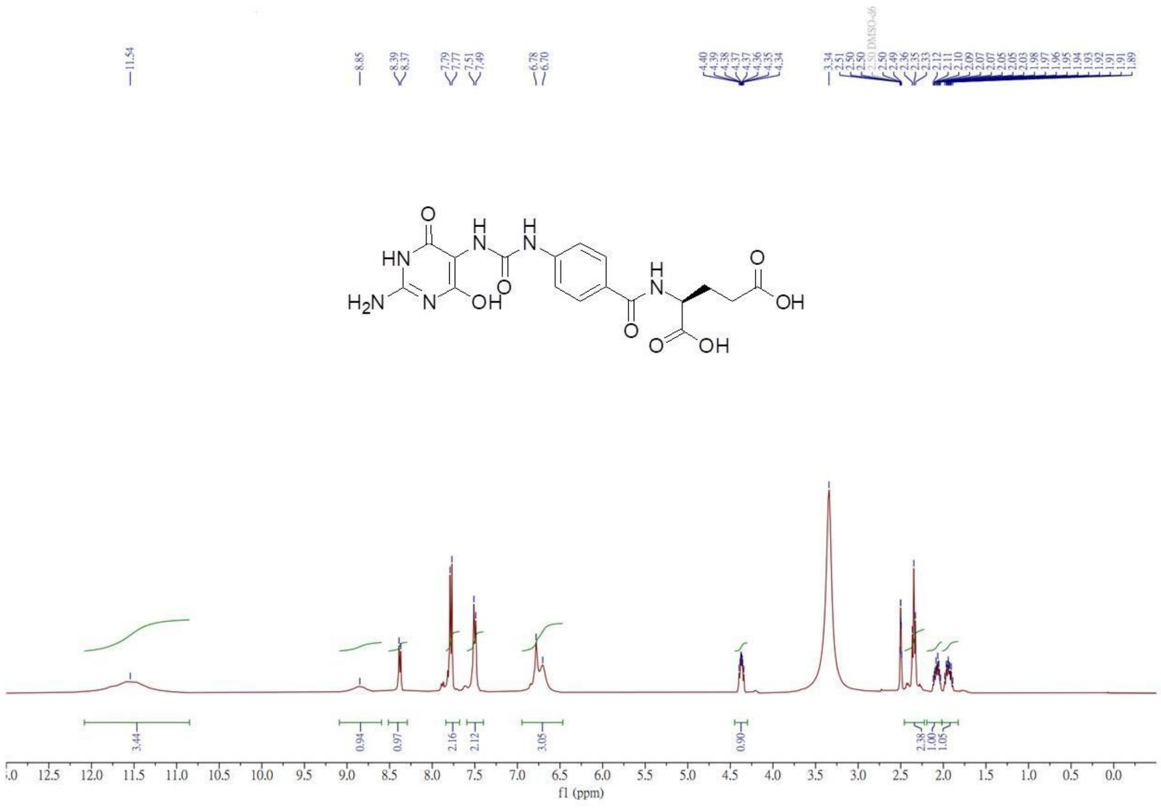

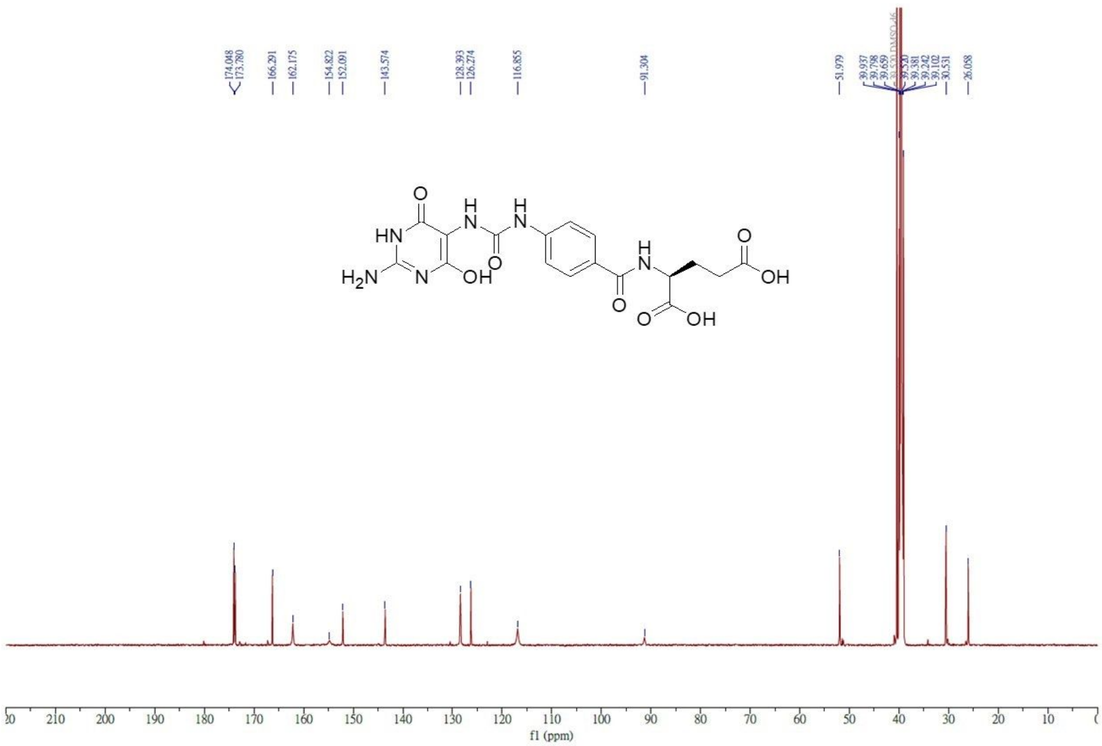

Figure S-12. ^{1}H and ^{13}C NMR spectrum of **16i** in DMSO-d$_6$.

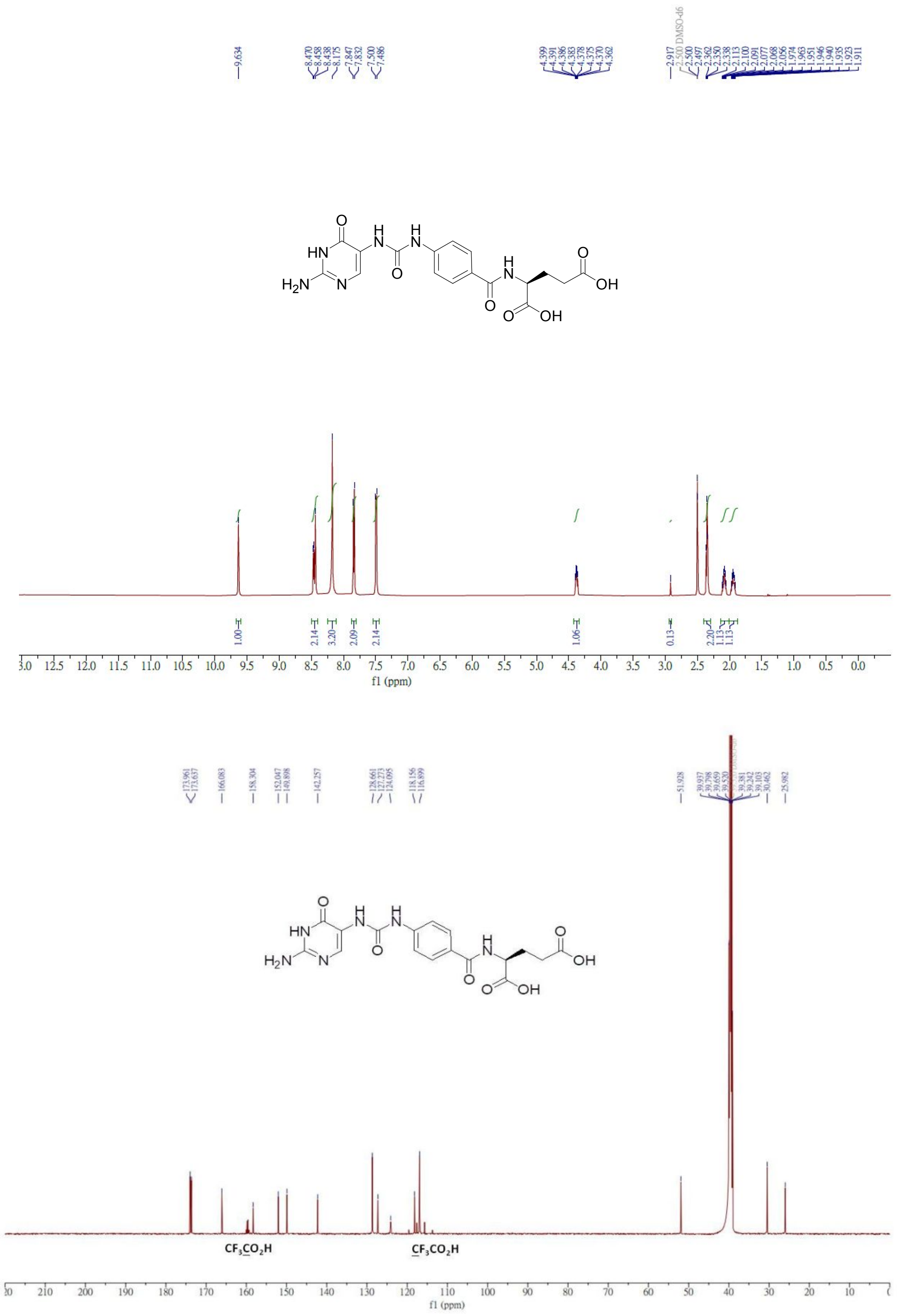

Figure S-13. ^{1}H and ^{13}C NMR spectrum of **16j** in DMSO-d_6.

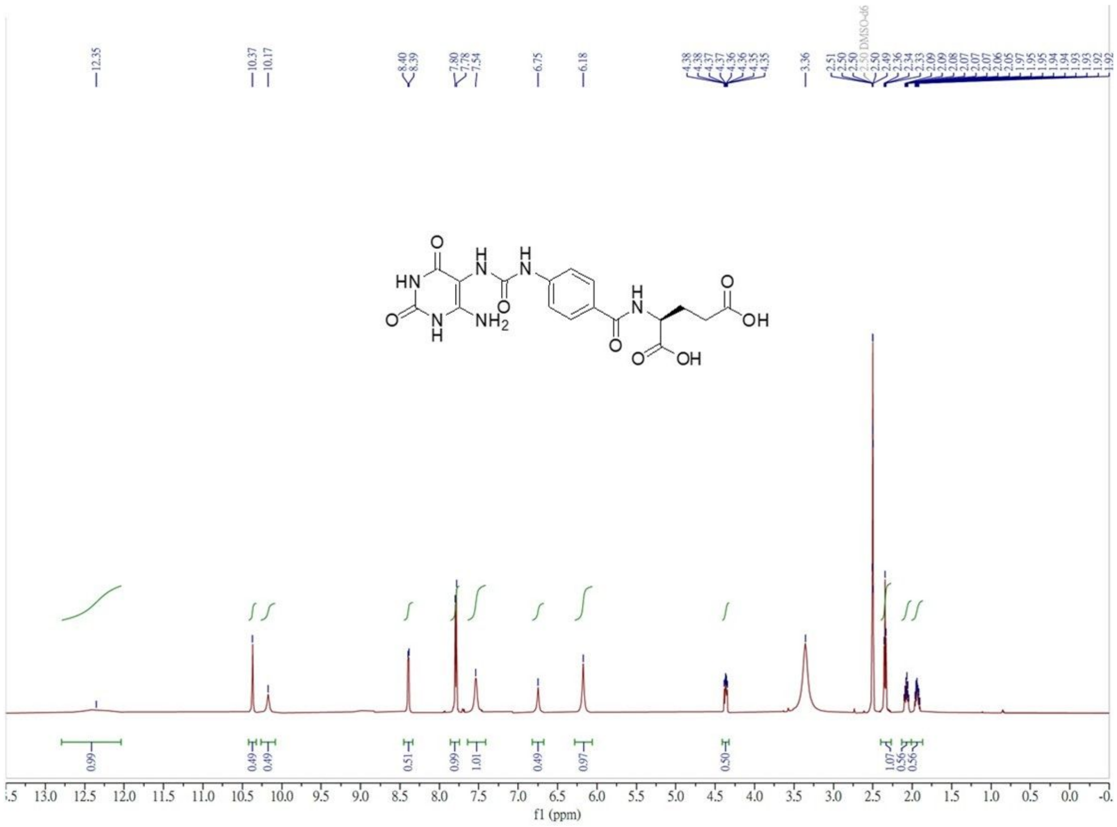

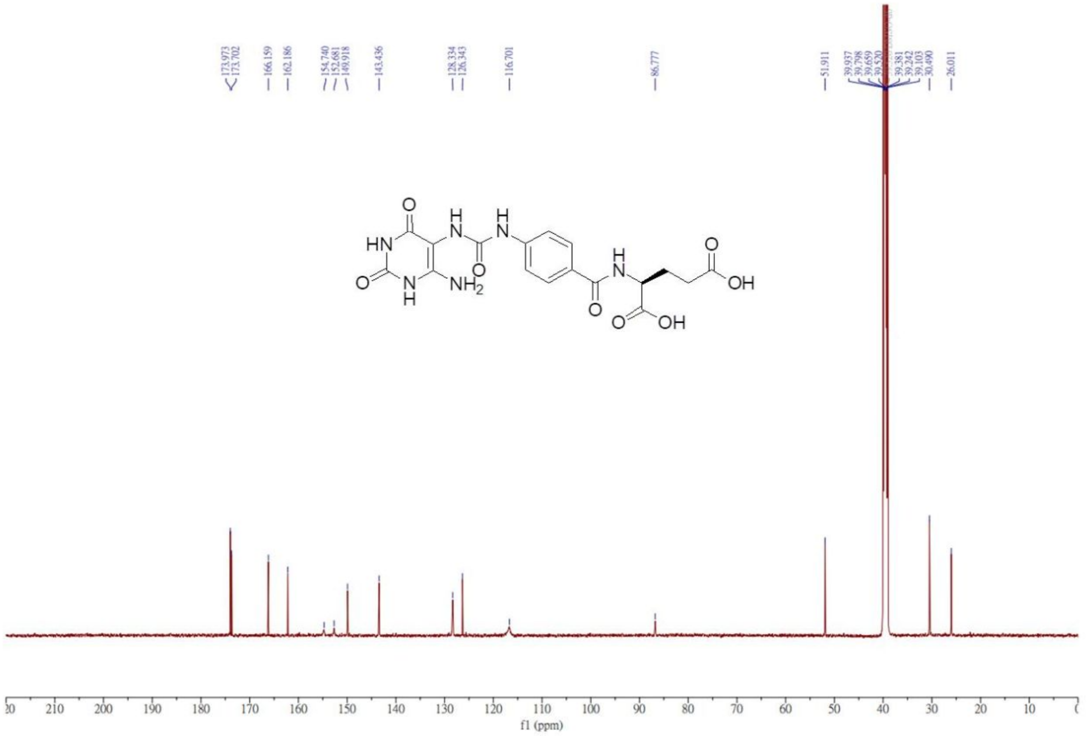

Figure S-14. ^{1}H and ^{13}C NMR spectrum of **16k** in DMSO-d_6.

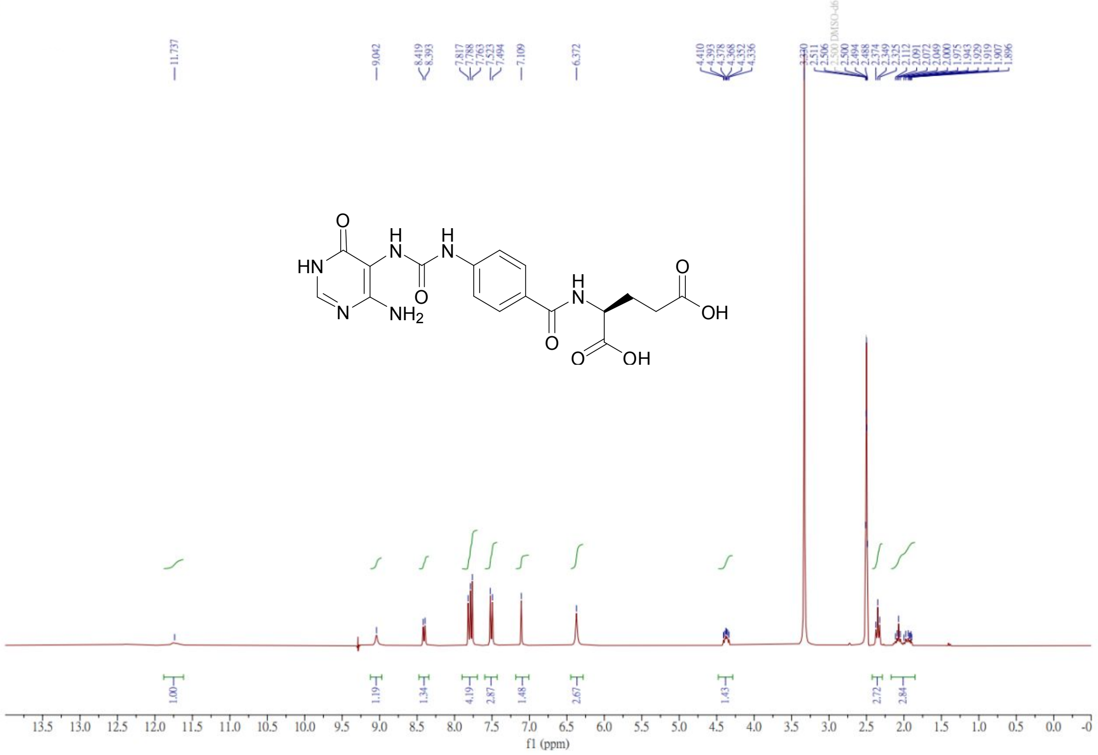

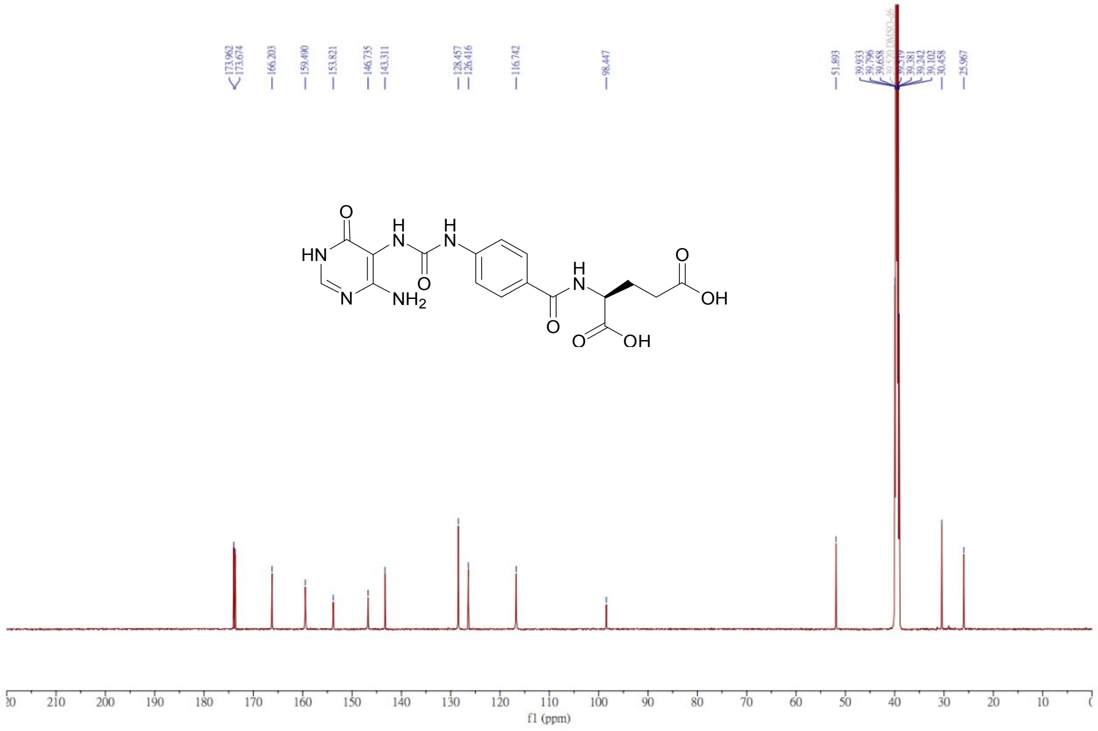

Figure S-15. ^{1}H and ^{13}C NMR spectrum of **16l** in DMSO-d_6.

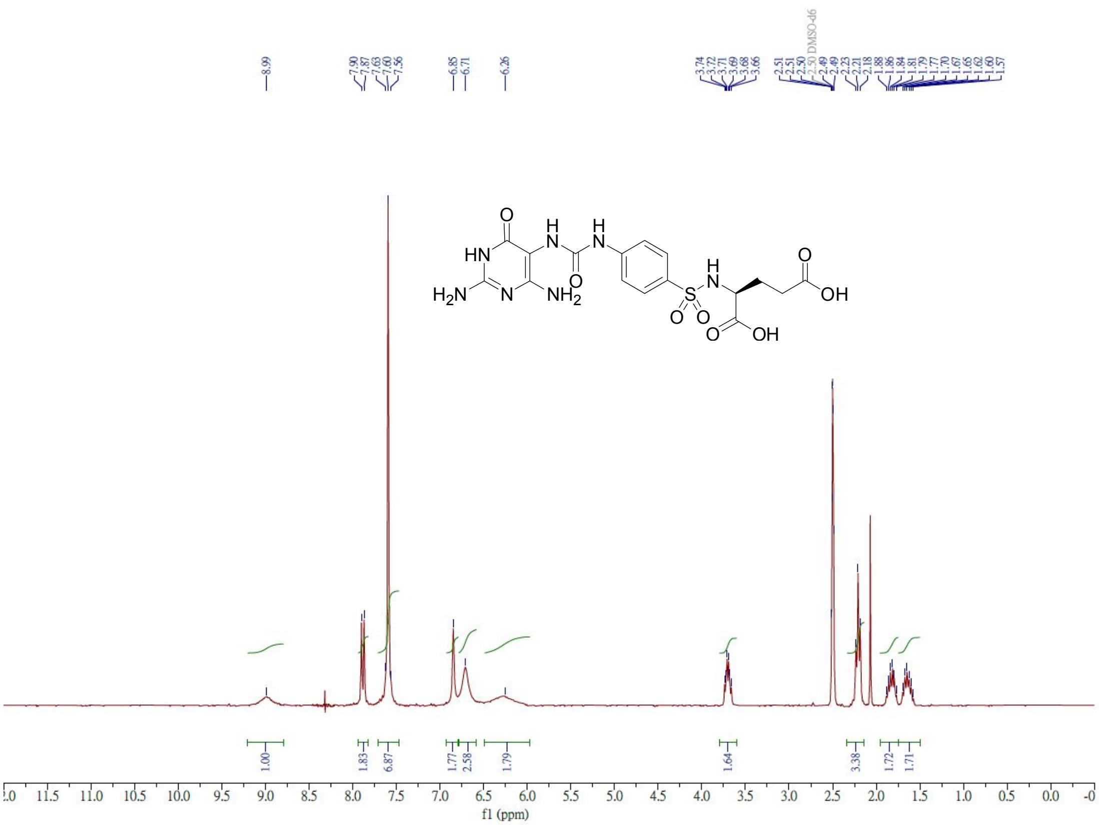

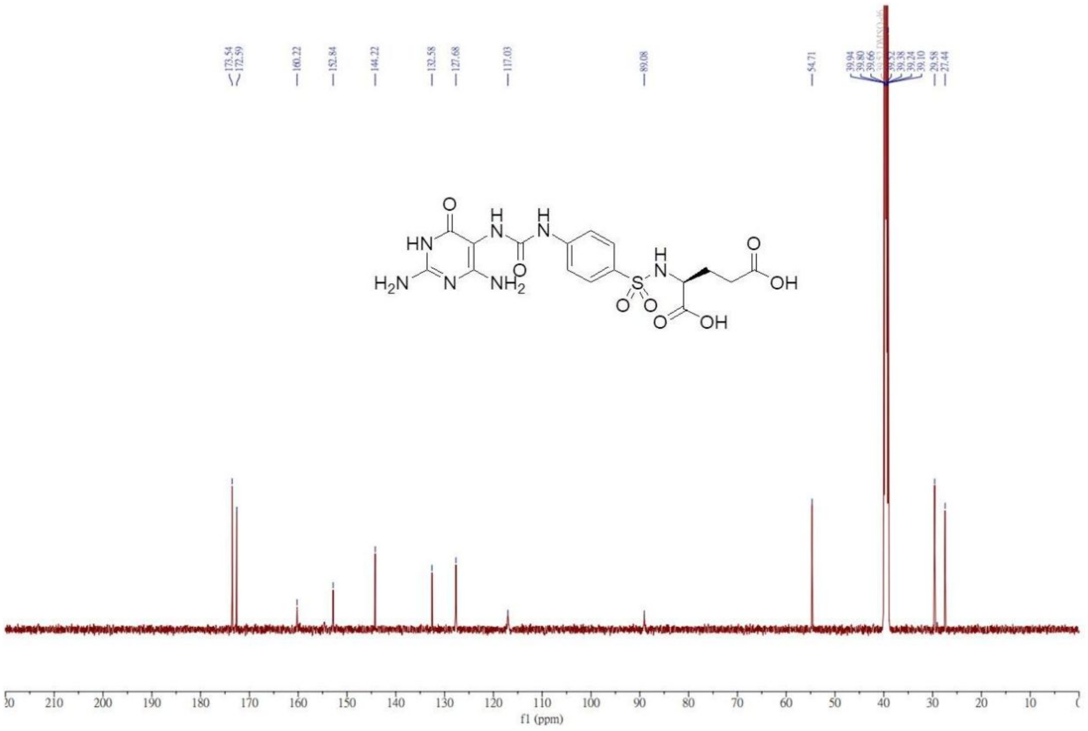

Figure S-16. ^{1}H and ^{13}C NMR spectrum of **19** in DMSO-d_6.

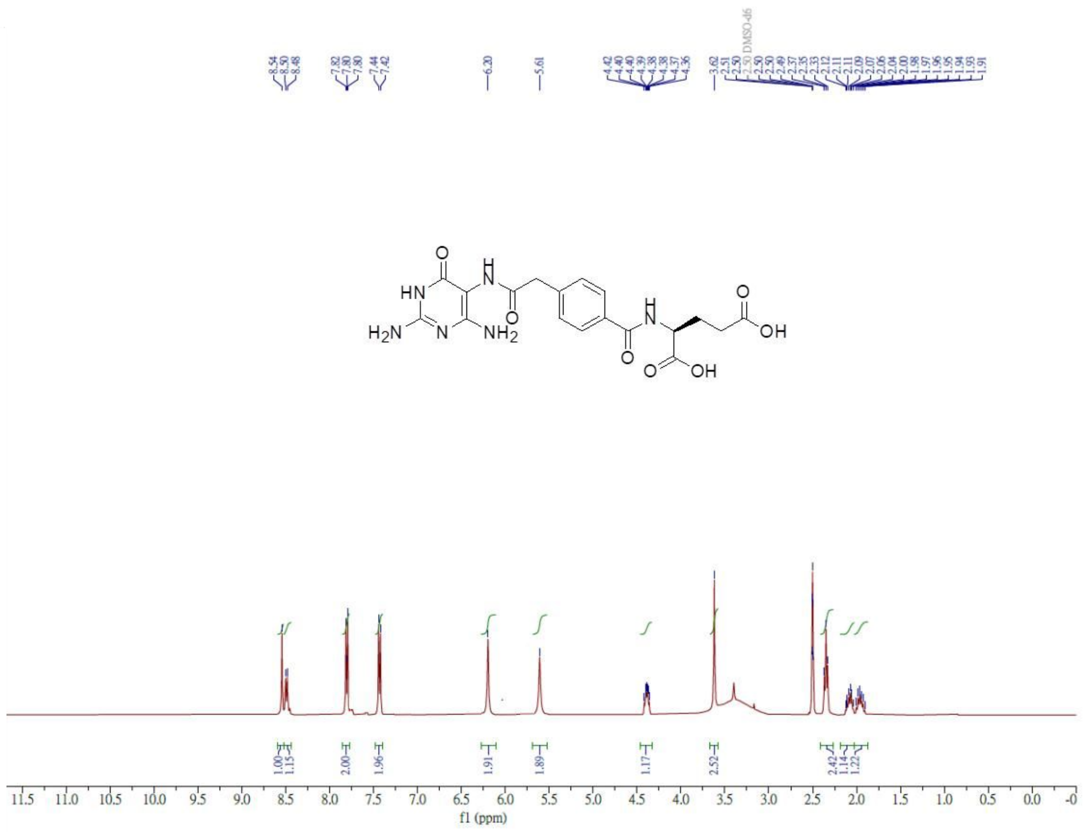

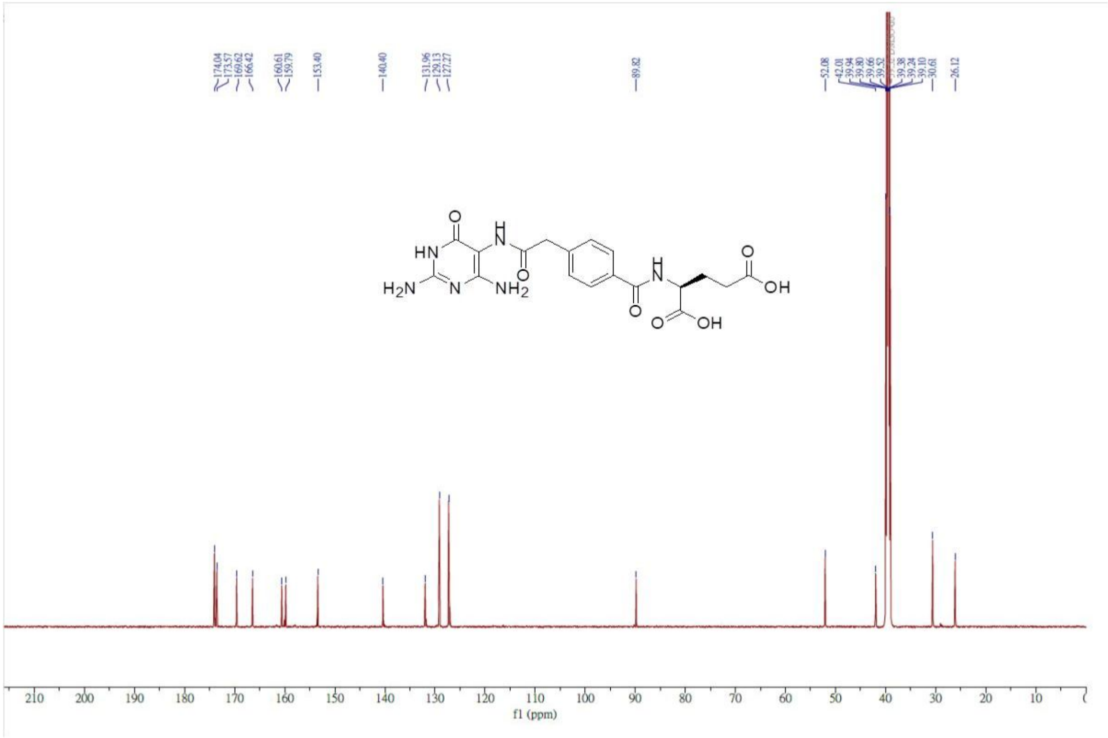

Figure S-17. ^{1}H and ^{13}C NMR spectrum of **23** in DMSO-d_6.
